# Pushing and Pulling: The Static and Dynamic Effects of Political Distrust on Support for Representative Democracy and its Rivals

**DOI:** 10.1007/s11109-024-09994-y

**Published:** 2025-01-10

**Authors:** Tom W. G. van der Meer, Lisa A. Janssen

**Affiliations:** 1 Department of Political Science, University of Amsterdam, Amsterdam, The Netherlands; 2 Department of Political Science, Ghent University, Ghent, Belgium

**Keywords:** Political trust, Panel data, Representative democracy, Direct democracy, Authoritarianism, Technocracy

## Abstract

Distrust is widely argued to stimulate support for political and institutional change. Yet, there is little agreement among scholars whether distrust pulls people towards rivaling decision-making models such as direct democracy, technocracy, and authoritarianism. This paper argues that political distrust is an unconditional push-factor away from the status quo (i.e., representative democracy), but that the appeal of any specific alternative decision-making models among distrusters is conditional on their political dispositions. This paper systematically tests rivaling theories on the micro-level relationship between political distrust and support for change, representative democracy, and alternative decision-making models. Crucially, we test to what extent the pull-factor of rivaling models is conditional on citizens’ political efficacy and populist leaning. Moreover, we separate the effects of structurally low trust from that of dynamically declining trust by estimating Random Effects Within Between (REWB) models on three-wave panel survey data across four European countries (the UK, the Netherlands, Sweden, and Portugal). Our findings confirm that distrust unconditionally pushes people away from the status quo, but does not unconditionally pull people towards any alternative model. Rather than technocracy (mixed effects) and authoritarianism (predominantly negative effects), we find that political distrust particularly stimulates support for direct democracy. This positive effect of political distrust on support for direct democracy is particularly strong among efficacious citizens and supporters of populist parties. This aligns with the idea of dissatisfied democrats, whose distrust drives their ambition for more direct influence.

**Supplementary Information:**

The online version contains supplementary material available at 10.1007/s11109-024-09994-y.

## Introduction

We could describe what is going on at the moment as a crisis of democracy, the collapse of trust: the belief that our leaders are not just corrupt or stupid, but inept. Action requires power, to be able to do things, and we need politics, which is the ability to decide what needs to be done. But that marriage between power and politics in the hands of the nation state has ended. Power has been globalized, but politics is as local as before. Politics has had its hands cut off. People no longer believe in the democratic system because it doesn’t keep its promises. (…) The current crisis of democracy is a crisis of democratic institutions.

Zygmunt Bauman, *El Pais*, 25 January 2016.

A longstanding theory in political trust research resonates in the public debate on the state of democracy. That theory reads that high levels of distrust undermine support for representative democracy and stimulate support for alternative decision-making models (Dalton, 2004) such as direct democracy (Ouattara & Van der Meer, 2023), technocracy (Bertsou & Caramani, 2020) and authoritarianism (Mounk, 2018). Ultimately, longstanding distrust could thereby atrophy democracy (Crozier et al., 1975) and/or induce “far reaching systemic change within the general category of representative democracies” (Fuchs & Klingemann, 1995: 7; see also Kaase & Newton, 1995: 30). This specter has hung over political trust research for decades.

Yet, the theorized causal effect of political distrust on support for representative democracy and its alternatives is not well developed theoretically or evident empirically. While there is relatively broad agreement that political distrust induces support for political change, this change may reflect diminished support for the status quo of representative democracy and/or increased support for any alternative model of decision-making. Political distrust has a push-factor (away from the status quo) and is often assumed to have a pull-factor (towards any specific alternative). The scholarly literature has put forward rivaling claims about the extent to which distrust pulls people towards alternative decision-making models. Moreover, systemic evidence on the consequences of political distrust is surprisingly scarce (Norris, 1999: p25; Dalton, 2004: 162; Torcal & Lago, 2066: 309; Van der Meer & Zmerli, 2017): “[M]ost of this debate is being conducted in the absence of reliable knowledge about the possible social and political consequences of lower levels of political trust” (Marien & Hooghe, 2011: 268).

The lack of empirical consensus on whether and how political distrust relates to support for representative democracy and its alternatives stems from several problems within the academic literature that his paper aims to address.

On the one hand, theories of political (dis)trust have not systematically distinguished between its push- and pull-factors. To the extent that political distrust induces a rejection of the status quo of representative democracy (Hibbing & Theiss-Morse, 2002; Caramani, 2020), we may expect that citizens become open to alternative models. However, political trust theory does not inform us which of these alternative models citizens will embrace. Rather, the literature has assumed theoretically and methodologically that the effects of political distrust on support for rivalling decision-making models are uniform across citizens. Yet, to the extent that alternative models appeal to distrusting voters, such pull-effects likely depend on citizens’ pre-existing political dispositions. We argue that political distrust is a push factor away from the status quo, but not necessarily a pull factor towards any specific alternative model. Which decision-making model distrusting citizens become attached to likely depends on their political dispositions. Hence, this paper will test to what extent the effect of political distrust on support for rival decision-making models is contingent on citizens’ internal efficacy and populist leaning.

On the other hand, the literature suffers from methodological limitations that directly impact the test of the theoretical argument that distrust conditionally pushes away from the status quo and towards alternative models. First, although they are better equipped to deal with causal questions, experimental and panel designs remained remarkably scarce. Rather, studies have predominantly assessed the correlates of levels of distrust at one point in time. Yet, the effects of high and rising distrust are likely to diverge, and have different theoretical implications (Ouattara & Van der Meer, 2023). This paper addresses this limitation by differentiating between the static effects of structurally high levels of political distrust and the dynamic effects of rising political distrust. Second, most studies have taken place in a single country, thereby overlooking the risk that country-specific political histories and contexts may affect the ways political distrust is expressed. This paper, therefore, tests the effects of political distrust in four West European countries selected for their divergence along two dimensions (high vs. low trust societies; majoritarian vs. proportional electoral system). Third, while various studies assessed the effect of political distrust on support for a specific decision-making model or institution, only few tested these effects more systematically on an agreed broad range of decision-making models simultaneously (cf. König et al., 2022). Because it is crucial to understand the push- and pull-factor of political distrust, this paper systematically tests its effects on a range of decision-making models (i.e., representative democracy, direct democracy, technocracy, and authoritarianism).

These theoretical and methodological considerations inform our research question: To what extent and under which conditions are high and rising political distrust related to support for representative democracy and rivaling decision-making models (direct democracy, technocracy, and authoritarianism)? This paper contributes to the literature in three key ways: (1) by consistently theorizing and testing the conditions (moderators) under which distrust pushes people away from the status quo and towards alternative models; (2) by employing panel data across four countries; and (3) by focusing on a broad range of decision-making models.

## Theory and Hypotheses

### Support for Change

Political trust is typically a ‘middle range indicator’ of political support aimed at the institutions of democracy (cf. Zmerli et al. 2007), between specific support (for individual leaders or policies) and diffuse support (for the regime and its values). As such, low or rising political trust may stimulate people to push for a change of leadership as a whole or even of the system itself. Throughout the political trust literature, high levels of political distrust have been associated with a push for transformation and change of the status quo (cf. Miller, 1974; Kaase & Newton, 1995; Fuchs & Klingemann, 1995; Dalton, 2004; Bengtsson & Mattila, 2009). This may take the shape in the short term, at the micro-level, as the replacement of politicians with new or radical rivals (cf. Bélanger, 2017; Voogd et al., 2019) or in the long term, at the macro-level, as a “revolutionary alteration of the political and social system” (Miller, 1974: 951). This push for change of the status quo resonates with the conclusion by Hibbing and Theiss-Morse (2002: 77) that “people who are frustrated with current processes want to see the system changed in virtually any way possible. (…) Any change is better than the status quo.”

Hence, the first, most straightforward expectation reads that distrust is associated with a desire for political change:

H1. Political distrust (static or dynamic) stimulates support for change to the system or the politicians in that system.

## Direction of Change

While political distrust is widely acknowledged to stimulate support for political change, the literature is less consistent about the direction of these changes. Support for change may be due to lowered support for the model underlying the status quo (i.e., representative democracy), increased support for an alternative model, or both. Different strands of research have theorized about the direction of these changes. Various studies have tested the effects on different types of decision-making models. Yet, there is little firm agreement on these models. Despite different labels and different choices, we see four common models in the political trust literature (cf. Coffé & Michels, 2014; Font et al., 2015; Bertsou & Caramani, 2022; Ouattara & Van der Meer, 2023)^1^: Representative democracy, Direct democracy, Technocracy, and (democratic or non-democratic) Authoritarianism.

What most studies agree on, both theoretically and empirically, is that relatively high distrust relates to relatively lower levels of support for representative democracy. This specifies the idea we introduced above that political distrust induces a desire for political change, by decreasing the support for the status quo. To the extent that this is a push factor of political distrust, we would expect distrusters to be more likely to reject the status quo. When the status quo is defined as representative democracy, it makes sense that the rejection of the status quo is reduced support for the model of representative democracy. Indeed, the object of political trust conventionally consists of representative institutions such as parliament, government, and political parties. Caramani (2020) describes the rejection of the status quo as “the distrust of parties for their short-term, vote-seeking and particularistic nature.” Hence, we expect that distrust undermines support for representative democracy specifically.

H2. Political distrust (static or dynamic) erodes support for representative democracy.

Yet, the rejection of the status quo does not evidently entail support for a single alternative model of politics. Crozier et al. (1975) wrote in their influential report for the Trilateral Committee: “[W]ith all this dissatisfaction, no significant support has yet developed for any alternative image of how to organize the politics of a highly industrialized society. (…) The lack of confidence in democratic institutions is clearly exceeded by the lack of enthusiasm for any alternative set of institutions.” Rivaling theories induce rivaling expectations on the decision-making models as a pull-factor, by appealing to distrusters. Three theoretical models have resonated in the literature.

The first is the theory of alienated citizens, commonly associated with foundational work in the 1970s such as Finifter (1970) and Miller (1974), and more recent echoes (Mair, 2013; Mounk, 2018). It posits that distrust pushes people towards non-democratic rule. The second theory emphasizes critical citizens (or dissatisfied democrats) and is commonly associated with the work of Norris (1999) and Dalton (2004). This relates distrust to an ambition for more democracy. Norris (1999) summed up the core of the thesis, as critical citizens “feel that existing channels for participation fall short of democratic ideals, and who want to improve and reform the institutional mechanisms of representative democracy.” The third theory is stealth democracy, introduced by Hibbing and Theiss-Morse (2002). It relates distrust primarily to a push for technocratic politics and only secondarily to a wish for direct citizen intervention in politics if the need arises acutely. Stealth democrats consider the divisiveness and self-interest in strung-out democratic processes repulsive, and appeal to a not very pluralistic notion that politicians should just get things done. “Technocracy is the desire to take the ‘politics’ out of the policy-making process, and it therefore correlates with low levels of trust towards actors primarily pursuing their own interest instead of aiming for the good of a putative ‘whole’” (Bertsou & Caramani, 2020).

Whilst these theoretical perspectives propose clear expectations with regards to the direction of the effects of political distrust (i.e., declining political trust drives support for direct democracy, technocracy, and authoritarianism), the empirical evidence has been inconsistent. Regarding direct democracy, higher levels of political distrust tend to relate to positive views towards the direct involvement of citizens in politics (Bedock & Pilet, 2020; Bessen, 2020; Christensen, 2018; Ouattara & Van der Meer, 2023; cf. Gherghina & Geissel, 2019; Mohrenberg et al., 2021). Yet, other studies find no significant effect (Coffé & Michels, 2014; Bengtsson & Mattila, 2009), mixed effects (Donovan & Karp, 2006), or even outright negative effects (Bowler et al., 2007). Regarding technocracy, findings are even less consistent: some studies find that high distrust relates to support for expert rule (Bertsou, 2022; Bertsou & Pastorella, 2017), others that high distrust undermines that support (Ganuza & Font, 2020), and most found mixed effects, depending a.o. on the indicator of political support (Bengtsson & Mattila, 2009; Coffé & Michels, 2014; Costa-Lobo & McManus, 2020; Chiru and Enyedi, 2022). Finally, the effect of distrust on support for authoritarianism has been a less common object of empirical study. Although trends in political distrust may coincide with support for authoritarian rule (Mounk, 2018), there is no indication that the two are related at the individual level. Rather, recent studies found no or even negative effects of political distrust on support for authoritarianism (Hirsch, 2022; Ouattara & Van der Meer, 2023).

The three main theories in the literature thus provide three expectations. If they all hold simultaneously – i.e., if citizens become more supportive of all three models at the same time in the face of rising distrust -, that would suggest that distrust indiscriminately pushes citizens away from the status quo in favor of ‘anything else’, rather than raising the appeal (pull) of any specific alternative model.

H3. Political distrust (static or dynamic) stimulates support for (a) direct democracy, (b) technocracy, and (c) authoritarian modes of government.

## Conditionality of Directed Change

While the literature proposes various alternative decision-making models that might appeal to distrusters, it does not specify why any of these in particular would pull in political distrusters. Ultimately, we argue, the appeal of a specific model is likely to depend on distrusters’ political dispositions.

One of these dispositions is the internal political efficacy of respondents, i.e., their confidence that they are capable of understanding and influencing politics. Because of this confidence, we may expect efficacious citizens to strive for more direct influence on the decision-making process (cf. Gherghina & Geissel, 2020). Hence, when political distrust shies them away from supporting the status quo, efficacious citizens are more likely than non-efficacious citizens to support direct democracy (as it raises their influence) and less likely than non-efficacious citizens to support decision-making models that reduce their political influence, including technocracy and authoritarianism.

H4a. Political distrust is more likely to stimulate support for direct democracy when internal political efficacy is high.

H4b. Political distrust is more likely to stimulate support for technocracy when internal political efficacy is low.

H4c. Political distrust is more likely to stimulate support for authoritarianism when internal political efficacy is low.

Another disposition is made visible by citizens’ vote intentions. Some parties strive for direct democracy over decision-making by politicians, whereas others do not. In recent years, direct democracy has been associated with populist parties. At the heart of populism lies the notion of the good people who are opposed by a corrupted political elite (cf. Mudde, 2004). Populist parties tend to support a majoritarian conception of politics (Canovan, 1999) and -albeit contingently- to support referendums (Gherghina & Pilet, 2021a). Populist voters tend to support the introduction and use of referendums (cf. Mohrenberg et al., 2021), the outcomes of which they consider to be more legitimate (Werner & Jacobs, 2022). The more that citizens support parties that embrace a majoritarian, direct-democratic view of democracy, the more that high or rising distrust pushes them towards direct democracy.^2^

Given their majoritarian conception of democracy, the populism-technocracy and populism-authoritarianism relationships are more complex. On the one hand, populism entails skepticism towards elites, particularly when the populist party is not in government. On the other hand, it appreciates an anti-political and anti-pluralistic understanding of politics, for instance of politics that behaves in a businesslike manner. Hence, we only formulate a hypothesis on the conditioning effect of democratic populism on the effect of political distrust on support for direct democracy.

H5. Political distrust is more likely to stimulate support for direct democracy among citizens who lean towards supporting populist parties than among citizens who do not.

## Data, Operationalization, and Method

### Data

Our hypotheses place several demands on the data and method: micro-level panel data across multiple countries covering political trust, support for rivaling decision-making models, and political dispositions. For that purpose, we collected panel survey data with the sampling frame of Kantar, which is not a fresh random sample but is representative on demographic traits.^3^ Data collection took place in four countries that vary along two important dimensions: their common trust rates and the proportionality of their electoral system. Our study encompasses Sweden (high trust, moderate proportionality), Portugal (low trust, moderate proportionality), the United Kingdom (moderate trust, low proportionality), and the Netherlands (moderate trust, high proportionality). The nature of the systems may affect support for rivaling decision-making models. In proportional more than in majoritarian systems people may therefore support limitations on power (Heyne 2018). Additionally, recent experience with and political debate on direct democracy also varies across these countries: in the late 2010s referendums were more salient in the UK and the Netherlands than in Portugal and Sweden.

We collected the panel data in three waves over seven months. Wave 1 took place in September 2020, shortly after the main COVID19-rally round the flag. Respondents were recontacted to take part in a second wave (November 2020), and finally a third wave (January-March 2021). The downside of this timing is that findings might be somewhat contingent on the experience of the pandemic (cf. Lavezzolo et al., 2022; Hirsch, 2022), although any bias is likely to be small and ambiguous (see Appendix G for an extended discussion). A total of 8,328 respondents took part in at least one of the three waves; 6,497 (78%) took part in at least two of these waves.

### Dependent Variables

Our hypotheses call for two sets of dependent variables. First, we assess support for democratic change, either in the form of leadership or the regime itself. For that purpose, we constructed a survey question ‘Thinking about the way political decisions are made in this country, which comes closest to your view?’. We offered three answer options to this question in the following sequence: (1) we need to radically change the way we make political decisions, (2) we need to change the political leadership, but not radically change the way we make decisions, and (3) no radical changes are needed to either the political leadership or the way we make political decisions in this country.^4^

Support for various types of government is measured via a battery of statements of potential models that we introduce with the statement ‘There are different ways to govern a country. Looking at the different options described below, what do you think about each as a way of governing our country?’. The respondents were asked to indicate their level of agreement on a five-point scale ranging from (1) strongly disagree to (5) strongly agree. We allowed respondents to express support for multiple models.^5^ This enables us to test whether distrust is primarily a push factor (indiscriminately away from the status quo) or also a pull factor (towards any specific alternative).

Support for representative democracy is measured with two items: ‘Citizens should choose members of parliament and the parliament then makes decisions’ and ‘It is best to leave politics to elected professional politicians’. Because the correlation between the pair of items is low and Mokken scale analyses showed insufficient scale strength, we did not combine these items into one scale but estimated models for each item separately. Possibly, the differential emphasis on citizen delegation (in the first item) and professional politicians (in the second item) invokes different connotations.

Support for direct democracy is measured with the statements ‘Citizens, not elected officials, should vote directly on major national issues to decide what becomes law’ and ‘We should make as many political decisions as possible by referendum’. The two items form a strong scale (H > 0.6 in all countries) that we use for our analyses below.

Support for technocracy is measured with a single item: ‘Non-elected experts should make decisions according to what they think is best for the country’.

Support for authoritarianism is measured with two items: ‘A strong leader should be able to make decisions without interference from parliament or courts’ and ‘A single non-elected president should decide what’s best for the country’. While the second item is clearly undemocratic, the first is not incompatible with elections. The two items form a strong Mokken scale (H > .5 in all countries) and are thus combined in our explanatory analyses.

## Independent Variables and Moderators

Political distrust is measured for various political institutions: government, parliament, courts, police, politicians, and political parties. Participants were asked to evaluate the trustworthiness of these institutions on a 5-point scale ranging from 1 (Strongly distrust) to 5 (Strongly trust). We recoded all variables so that high scores signal more distrustful evaluations. Mokken scale analyses confirmed that the items can be combined into a strong scale for all countries and waves (H ≥ 0.60). The average trust in the six institutions measures the respondents’ general political distrust.

Hypotheses 4a-c emphasize internal political efficacy. We measured internal political efficacy on a scale from (1) strongly disagree to (5) strongly agree using the following two statements: ‘I feel I have a pretty good understanding of the important political issues facing our country’ and ‘I consider myself well qualified to participate in politics’. The combined items form a strong Mokken scale (H ≥ 0.52). We did not incorporate external political efficacy as a determinant, as that would risk endogeneity with the political distrust measure to which it conceptually relates (cf. Van der Meer, 2017).

Hypothesis 5 requires a measure of preference for populist parties. We measure party preference with the question ‘Currently, which political party appeals to you most?’, followed by a standardized list of parties represented in parliament. Because a dichotomous understanding of populism (cf. Rooduijn et al., 2019) would not discriminate sufficiently for the purposes of our study, we rely on the Chapel Hill Expert Survey (Bakker et al. 2020) to establish the degree to which the political parties can be regarded as populist on a people-versus-elite scale. The 2019 wave is the last pre-pandemic wave, collected in the Winter of 2020. Experts assigned political parties scores between (0) ‘Elected office holders should make the most important decisions’ and (10) ‘The people, not politicians, should make the most important decisions’. We calculate the respondent’s overall leaning by taking the average score on the people-versus-elite measure over the waves. We thus only model populist leaning as time-invariant and not as a time-variant trait: as a time-variant trait, the model would be too noisy, particularly as the share of within-person variance (16%) is very small for panel data. Unfortunately, the data on Portugal face a limitation: the measures do not cover the populist party Chega, which was erected in 2019, or Liberal Initiative, which was erected in 2017. In line with the polls at that time, a substantial share of respondents considered voting for these parties. This likely makes our findings somewhat conservative.

Conceptually and methodologically, political distrust taps into only one of the three aspects of populism (anti-elitism) but not into people-centrism or the inherent antagonism between elites and the people (which our CHES measure emphasizes) (cf. Geurkink et al., 2020). Our measures of distrust and populism have a moderately strong relationship (0.36). Scatterplots illustrate a spread of respondents across all quadrants. There are distrusting citizens that score high on the people-versus-elite scale of populism, and distrusting citizens that do not. Similarly, some citizens who think that people should make the most important decisions tend to distrust politics, whereas others tend to trust politics.

We control for various demographic variables: age (in years), gender, and education level (standardized). To isolate the effect of political trust from the general effect of support for democracy, we control for the importance that respondents attribute to living in a democracy. This variable is measured with the question ‘How important is it for you to live in a country that is governed democratically?’ on a 7-point scale ranging from (1) not at all important to (7) very important. A robustness check shows that our findings are not affected by the inclusion of this control variable. Finally, we performed additional robustness checks that show that our findings are robust to the inclusion of support for government parties (see Appendix E).

To account for unobserved heterogeneity over the four countries in our data, we include country-level fixed effects. Appendix B reports separate analyses per country. We find rather strong country effects on the level of support for political change and decision-making processes. While some effects of political distrust are remarkably similar across countries, not all of them are. When they differ, we report that in the results section.

## Data Cleaning and Missing Values

We eliminated 1,831 respondents who only participated in a single wave, as our hypotheses require us to pull apart static from dynamic effects. Another 324 respondents were eliminated for straightlining^6^ across multiple question batteries where that is theoretically unlikely. Finally, our analyses are based on the listwise deletion per model of respondents with missing values. A full overview of the number of participants by country is provided in Appendix D.

### Method

The Within-Between Random Effects (RE) framework (cf. Bell et al., 2019) allows us to disentangle the static effects of structural levels of political distrust (between-respondents) and the dynamic effects of changing levels of political distrust (within-respondents). This model is suitable for panel data with a hierarchical structure in which repeated observations (level 1) are nested in respondents (level 2). The Within-Between RE model explicitly estimates the heterogeneity on the respondent-level (allowing for the substantial interpretation of both time-varying and time-invariant variables) whereas a fixed-effects model would only control for that heterogeneity. The between-respondent effect of political distrust essentially constitutes the respondent’s average political distrust over the waves, whereas the within-respondent effect constitutes the longitudinal variations from this average for every participated wave.^7^ An advantage of this framework is that it satisfies the assumption that level-1 predictors are uncorrelated with the random effects term. Moreover, since we leverage three-wave panel data in combination with REWB models, we reduce endogeneity concerns driven by time-invariant confounders common in cross-sectional research. Nevertheless, ultimately we cannot methodologically rule out endogeneity concerns arising from the possibility of reversed causality.

Given the categorical nature of the dependent variable central to hypothesis 1, our first analysis takes the shape of a multinomial logistic regression model. To test the remaining hypotheses, we estimate linear regression models. Finally, when we estimate cross-level interaction effects (of within- and between-person determinants), we include a random slope of the lower-level variable (Heisig & Schaeffer, 2019). While methodologically the combinations of two levels of analysis allow four unique interaction effects of the same two determinants, theory offers no expectation at which level the interaction should take place. Out of parsimony, we therefore only model the most straightforward interaction effects, i.e. the between*between interaction (for both moderators), within*within (for internal efficacy), and between*within (for populism).

### Results I: Direct Effects

Table 1 shows the results of the multinominal analysis of support for political change. Support for changes to political leadership or to decision-making processes is lowest in the Netherlands, and highest in Portugal. There are consistent effects of political distrust at the within- and between-levels. People with structurally higher levels of distrust are more likely to prefer a change of leadership (b = 2.1) and a change of procedures (b = 2.7). Similarly, when political distrust increases over time, people are more likely to prefer leadership change (b = 0.9) and procedural change (b = 1.2). We find support for H1.

**Table 1:** Support for political change by political distrust, REWB models

	Change of leadership	Change of process
*Within*		
Political distrust	0.894^***^	1.173^***^
	(0.055)	(0.060)
Importance of democracy	−0.236^***^	−0.294^***^
	(0.046)	(0.048)
*Between*		
Political distrust	2.124^***^	2.729^***^
	(0.068)	(0.071)
Importance of democracy	−0.475^***^	−0.621^***^
	(0.055)	(0.056)
*Country (ref: UK)*		
NL	−1.799^***^	−1.506^***^
	(0.106)	(0.109)
SE	0.193	−0.810^***^
	(0.106)	(0.116)
PT	0.901^***^	0.964^***^
	(0.126)	(0.130)
*Constant*	*−1.587* ^***^	*−3.499* ^***^
	*(0.420)*	*(0.434)*

Unstandardized b-values; Standard errors in parentheses

^+^*p* < 0.10, ^*^p < 0.05, ^**^p < 0.01, ^***^p < 0.001

N = 17,064

Controls for gender, age, and level of education

Next, we move to the direction of support for rivalling decision-making models in Table 2. First, we consider the between-person effects of political distrust. People with structurally high levels of distrust are less likely to support representative democracy: respondents subscribe significantly less to this model, both when we formulate it as delegation to an elected parliament (b=−0.133) and (especially) when we formulate it as elected professional politicians (b=−0.459). These effects are highly robust across countries and support H2. Structural distrust stimulates support for direct democracy (b = 0.430), an effect that is consistent across the four countries (see appendix B). This supports H3a and suggests that distrust is at least in part a call for more or different democracy. However, Table 2 also shows a weak, positive effect of structural distrust on support for rule by unelected experts (b = 0.037). While the main effect supports H3b, additional analyses show that it is driven strongly by one country, Sweden. We do not find significant effects in the Netherlands and Portugal and even a negative effect in the United Kingdom (see Appendix B).

Finally, we find no significant effect of systematic distrust on support for authoritarian rule. This rejects H3c. Closer inspection reveals no significant effect for the item of electoral authoritarianism, and a negative effect for undemocratic authoritarianism (see Appendix A). The latter suggests that distrust relates to a rejection of undemocratic authoritarianism. However, there are cross-national differences (see Appendix B). While we find consistent negative effects in Portugal and the United Kingdom, systematic distrust is related to higher support for authoritarianism in Sweden.^8^

**Table 2:** Explaining support for decision-making processes by political distrust, REWB models

	Delegate by electing parliament	Elected professional politicians	Direct democracy	Non-elected expert rule	Authoritarian
*Within*					
Political distrust	−0.143^***^	−0.284^***^	0.097^***^	−0.076^***^	−0.115^***^
	(0.012)	(0.013)	(0.011)	(0.014)	(0.010)
Importance of democracy	0.078^***^	0.020^+^	−0.025^**^	−0.099^***^	−0.110^***^
	(0.009)	(0.010)	(0.009)	(0.011)	(0.008)
*Between*					
Political distrust	−0.133^***^	−0.459^***^	0.430^***^	0.037^**^	−0.017
	(0.011)	(0.012)	(0.014)	(0.014)	(0.012)
Importance of democracy	0.125^***^	0.001	−0.089^***^	−0.211^***^	−0.377^***^
	(0.011)	(0.012)	(0.013)	(0.013)	(0.011)
Country (ref: UK)					
NL	−0.398^***^	0.143^***^	−0.058^*^	−0.131^***^	−0.594^***^
	(0.023)	(0.025)	(0.029)	(0.028)	(0.023)
SE	−0.075^**^	−0.175^***^	0.062^*^	0.403^***^	−0.642^***^
	(0.024)	(0.026)	(0.030)	(0.029)	(0.024)
PT	−0.162^***^	−0.346^***^	0.392^***^	0.209^***^	−0.380^***^
	(0.026)	(0.029)	(0.033)	(0.032)	(0.027)
*Constant*	*3.443* ^***^	*4.781* ^***^	*2.409* ^***^	*4.001* ^***^	*4.825* ^***^
	*(0.086)*	*(0.094)*	*(0.108)*	*(0.105)*	*(0.088)*
L2 variation	0.219^***^	0.271^***^	0.515^***^	0.367^***^	0.298^***^
	(0.008)	(0.010)	(0.012)	(0.012)	(0.008)
L1 variation	0.511^***^	0.626^***^	0.430^***^	0.701^***^	0.393^***^
	(0.007)	(0.009)	(0.006)	(0.010)	(0.005)
Observations	16,394	16,685	16,852	16,520	16,922

Unstandardized b-values; Standard errors in parentheses

^+^*p* < 0.10, ^*^p < 0.05, ^**^p < 0.01, ^***^p < 0.001

Controls for gender, age, and level of education

Table 2 also shows the within-person effects of political distrust. As we theorized, when distrust goes up, support for representative democracy goes down, both in the formulation that emphasizes delegation to an elected parliament (b=−0.14) and especially in the formulation that emphasizes elected professional politicians (b=−0.28). This is consistent across countries (see appendix B) and in line with H2.

The within-person effects of political distrust differ for the three alternatives to representative democracy and are less consistent across countries (see appendix B). When distrust goes up, so does support for direct democracy (b = 0.10). Remarkably, rising distrust erodes support for non-elected expert rule (b=−0.08) and support for authoritarian rule (b=−0.12). Further analyses show that the negative effect on expert rule is only significant in the United Kingdom (see Appendix B), and that the negative effect on authoritarianism exists for electoral as well as undemocratic authoritarianism (see Appendix A), and exists in all countries (see Appendix B).

In sum, we find support for hypothesis 2 (representative democracy) and hypothesis 3a (direct democracy), mixed support for hypothesis 3b (expert rule), while we reject hypothesis 3c (authoritarianism). That distrust pushes citizens away not only from representative democracy but also from authoritarianism goes against longstanding claims in the political trust literature that rising distrust might fuel support for undemocratic modes of government. Rather, the effects of changing political trust rates are best in line with the model of critical citizens (or disaffected democrats) who look for more influence on politics (cf. Norris, 1999; Dalton, 2004).

Support for non-elected expert rule is quite interesting. Whereas structural distrust stimulates support for non-elected experts, rising distrust more strongly diminishes that support. Methodologically, this paradoxical finding supports the distinction between levels and changes. Theoretically, it suggests that structural distrust may be more in line with the model of alienated citizens whereas dynamic distrust is more in line with that of critical citizens who respond to changes (cf. Ouattara & Van der Meer 2023).

### Results II: Conditional Effects

**Fig. 1:**
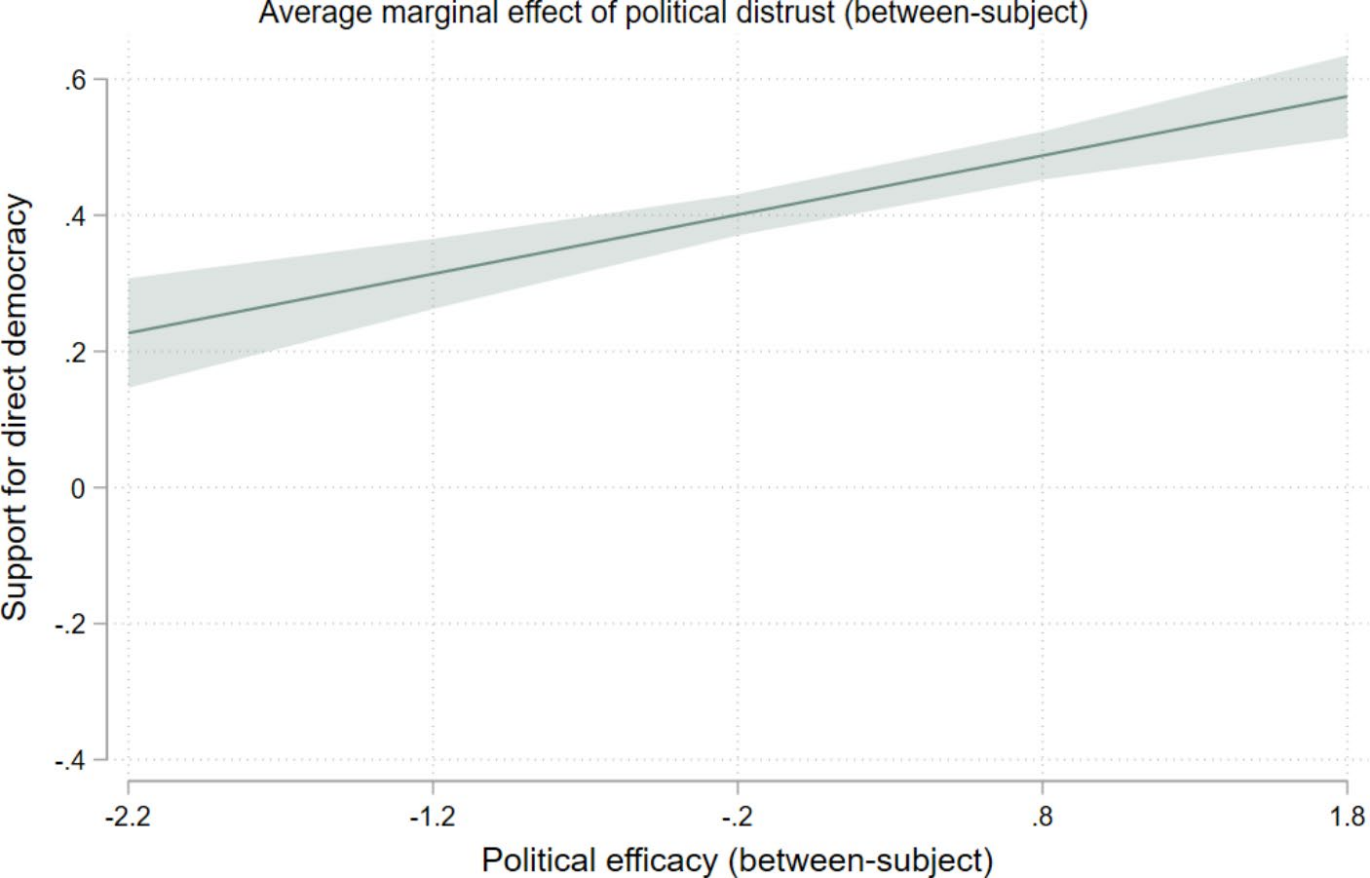
The average marginal effect of political distrust (between-subject) by political efficacy

Hypotheses 4a and 4b formulated the expectation that, paired with high levels of internal political efficacy, distrusters are more likely to support direct democracy and less likely to support non-elected expert rule. We find evidence for the former but not for the latter (see Table 3). Internal efficacy strengthens the between-person effect of political distrust on support for direct democracy (b = 0.09) but not the within-person effect. Figure 1 visualizes the marginal effects of political distrust on support for direct democracy, depending on the level of internal efficacy. In line with hypothesis H4a, the ascending slope in the figure illustrates that political distrust more strongly stimulates support for direct democracy when respondents’ level of internal political efficacy is higher. All in all, H4a finds support.

**Table 3:** The conditional effect of political distrust on support for decision-making processes, by internal efficacy

	Delegate by electing parliament	Elected professional politicians	Direct democracy	Non-elected expert rule	Authoritarian
*Within*					
Political distrust	−0.138^***^	−0.282^***^	0.099^***^	−0.075^***^	−0.116^***^
	(0.012)	(0.013)	(0.011)	(0.014)	(0.010)
Importance of democracy	0.073^***^	0.024^*^	−0.028^**^	−0.098^***^	−0.107^***^
	(0.010)	(0.011)	(0.009)	(0.011)	(0.008)
Internal efficacy	0.084^***^	−0.017	0.044^***^	−0.003	−0.019^+^
	(0.013)	(0.014)	(0.012)	(0.015)	(0.011)
*Between*					
Political distrust	−0.111^***^	−0.458^***^	0.418^***^	0.031^*^	−0.020^+^
	(0.011)	(0.013)	(0.014)	(0.014)	(0.012)
Importance of democracy	0.108^***^	0.021^+^	−0.090^***^	−0.197^***^	−0.360^***^
	(0.011)	(0.012)	(0.014)	(0.013)	(0.011)
Internal efficacy	0.433^***^	−0.046	−0.274^***^	−0.148^**^	−0.126^**^
	(0.039)	(0.044)	(0.051)	(0.049)	(0.041)
*Interaction*					
Political distrust (within)	−0.027	−0.075^**^	−0.018	−0.037	−0.044^+^
* Internal efficacy (within)	(0.026)	(0.029)	(0.027)	(0.031)	(0.024)
Political distrust (between)	−0.095^***^	−0.023	0.087^***^	0.020	0.008
* Internal efficacy (between)	(0.013)	(0.014)	(0.016)	(0.016)	(0.013)
*Constant*	*3.452* ^***^	*4.658* ^***^	*2.457* ^***^	*3.941* ^***^	*4.740* ^***^
	*(0.086)*	*(0.095)*	*(0.110)*	*(0.107)*	*(0.088)*
L2 variation	0.206^***^	0.264^***^	0.511^***^	0.362^***^	0.291^***^
	(0.008)	(0.010)	(0.012)	(0.012)	(0.008)
L1 variation	0.508^***^	0.626^***^	0.430^***^	0.701^***^	0.392^***^
	(0.007)	(0.009)	(0.006)	(0.010)	(0.005)
Observations	16,345	16,633	16,799	16,476	16,869

Unstandardized b-values; Standard errors in parentheses

^+^*p* < 0.10, ^*^p < 0.05, ^**^p < 0.01, ^***^p < 0.001

Controls for gender, age, level of education, and country

While we find a negative between-person effect of internal efficacy on support for expert rule (suggesting that efficacious people are less supportive of that model of decision-making), neither of the interaction effects is significant. We therefore reject H4b. Similarly, we do not find the expected interaction effect between internal efficacy and political distrust on support for authoritarianism.^9^ Hence, we reject H4c.

Although we had not theorized about the effect, Table 3 also shows that the negative effects of political distrust on support for representative democracy are stronger among efficacious people: there is a significant between-person interaction on delegation to an elected parliament (b=−0.10) and a significant within-person interaction on elected professional politicians (b=−0.08).

**Table 4:** The conditional effect of political distrust on support for decision-making processes, by populist party leaning

	Delegate by electing parliament	Elected professional politicians	Direct democracy	Non-elected expert rule	Authoritarian
*Within*					
Political distrust	−0.170^***^	−0.225^***^	0.067	0.037	−0.056
	(0.042)	(0.048)	(0.045)	(0.049)	(0.042)
Importance of democracy	0.079^***^	0.015	−0.036^***^	−0.118^***^	−0.109^***^
	(0.011)	(0.012)	(0.010)	(0.012)	(0.009)
*Between*					
Political distrust	−0.307^***^	−0.306^***^	0.263^***^	0.138^***^	0.031
	(0.031)	(0.034)	(0.039)	(0.039)	(0.033)
Importance of democracy	0.136^***^	−0.013	−0.108^***^	−0.228^***^	−0.401^***^
	(0.012)	(0.013)	(0.015)	(0.015)	(0.013)
Populist party leaning	−0.055^**^	0.031	0.044^+^	0.078^**^	0.028
	(0.020)	(0.022)	(0.025)	(0.025)	(0.021)
*Interaction*					
Political distrust (within)	0.003	−0.014	0.010	−0.026^*^	−0.015^+^
* Pop. party leaning (between)	(0.009)	(0.010)	(0.010)	(0.011)	(0.009)
Political distrust (between)	0.032^***^	−0.024^***^	0.019^*^	−0.024^**^	−0.010
* Pop. party leaning (between)	(0.006)	(0.007)	(0.008)	(0.008)	(0.007)
*Constant*	*3.732* ^***^	*4.626* ^***^	*2.515* ^***^	*3.804* ^***^	*4.815* ^***^
	*(0.129)*	*(0.141)*	*(0.164)*	*(0.162)*	*(0.136)*
L2 variation	0.090^***^	0.158^***^	0.297^***^	0.146^***^	0.241^***^
	(0.013)	(0.020)	(0.021)	(0.021)	(0.019)
L1 variation	0.217^***^	0.265^***^	0.517^***^	0.384^***^	0.325^***^
	(0.008)	(0.010)	(0.013)	(0.013)	(0.009)
Random slope	0.473^***^	0.579^***^	0.348^***^	0.648^***^	0.332^***^
political distrust (within)	(0.008)	(0.010)	(0.006)	(0.011)	(0.006)
Observations	13,938	14,139	14,268	14,036	14,321

Unstandardized b-values; Standard errors in parentheses

^+^*p* < 0.10, ^*^p < 0.05, ^**^p < 0.01, ^***^p < 0.001

Controls for gender, age, level of education, and country

**Fig. 2:**
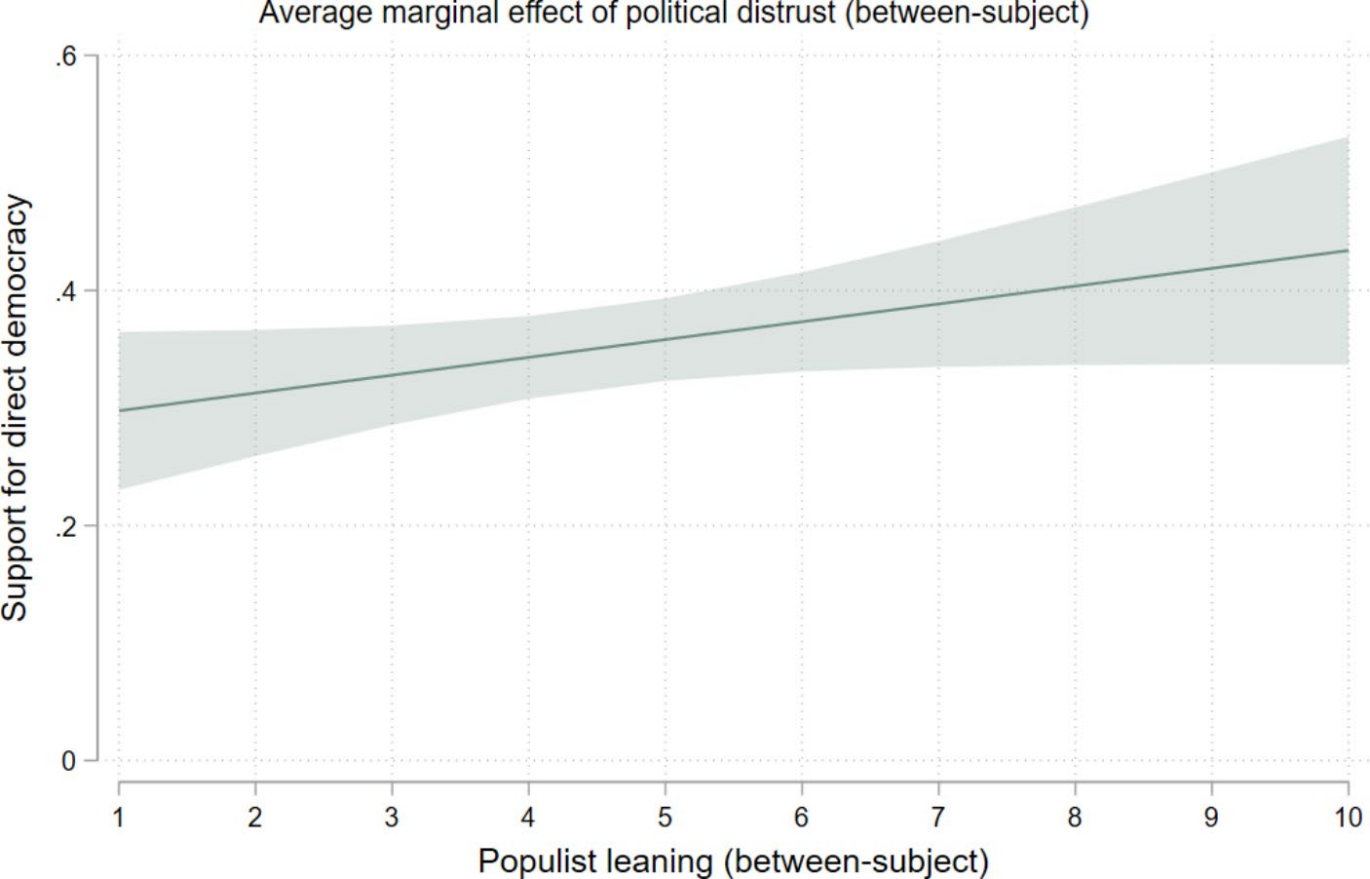
The average marginal effect of political distrust (between-subject) by populist leaning

Finally, we turn to the conditioning effect of populist party leaning, which we only measured as a between-person trait. We had expected that populist party leanings would stimulate the effect of political distrust on support for direct democracy. This is indeed what we find at the between-person level (see Table 4). As evidenced by the ascending lines in the marginal effects plots in Fig. 2, systematic distrusters are particularly more likely to support direct democracy when they lean populist in their vote intentions than when they do not (b = 0.02). Concurrently, an equally valid interpretation of this interaction effect reads that populist leaning citizens tend to support direct democracy, particularly when they also distrust politics. In other words, the pull-factor of direct democracy is especially strong among populist leaning voters in combination with the push-factor away from the status quo. This is in line with hypothesis 5.^10^ However, we do not find a significant interaction at the within-person level, i.e., for changing levels of political distrust.

Intriguingly, and although we had not theorized about that, we also find conditional effects on other modes of decision-making. The populist party leaning weakens the between-person effect of systematic distrust on one measure of representative democracy (voters delegating to an elected parliament) but strengthens the between-person effect of systematic distrust on the other (decisions by elected professional politicians). One potential explanation for this differential effect is that the first measure emphasizes the role of voters (a boon in populist rhetoric), whereas the second emphasizes professional politicians (which populist rhetoric considers to be corrupted; cf. Mudde, 2004). Moreover, populist leanings turn the within- and between-person effects of political distrust on non-elected expert rule more negative. These effects are visualized in Appendix C.

Table 5 provides an overview of all findings.

**Table 5:** Overview of findings

	Conclusion	Estimated effect				
	Full data	Full data	NL	UK	SE	PT
H1: Political distrust stimulates support for change to the system or the politicians in that system. - Between-respondent effect - Within-respondent effect	Supported	+ +	+ +	+ +	+ +	+ +
H2: Political distrust erodes support for representative democracy. - Between-respondent effect - Within-respondent effect	Supported	- -	n.s./- -	- -	- -	- -
H3: Political distrust stimulates support for:						
a) Direct democracy - Between-respondent effect - Within-respondent effect	Supported	+ +	+ n.s.	+ +	+ n.s.	+ n.s.
b) Technocracy - Between-respondent effect - Within-respondent effect	Mixed	+ -	n.s. n.s.	- -	+ n.s.	n.s. n.s.
c) Authoritarianism - Between-respondent effect - Within-respondent effect	Rejected	n.s. -	n.s. -	- -	+ n.s.	- -
H4: Political distrust is more likely to stimulate support for:						
a) Direct democracy when efficacy is high	Supported					
- Between-respondent effect		+	+	n.s.	+	+
- Within-respondent effect		n.s.	n.s.	n.s.	n.s.	n.s.
b) Technocracy when efficacy is low	Rejected					
- Between-respondent effect		n.s.	n.s.	-	+	n.s.
- Within-respondent effect		n.s.	n.s.	n.s.	n.s.	n.s.
c) Authoritarianism when efficacy is low	Rejected					
- Between-respondent effect		n.s.	n.s.	-	n.s.	n.s.
- Within-respondent effect		n.s.	n.s.	n.s.	n.s.	n.s.
H5: Political distrust stimulates support for direct democracy among populist voters	Supported					
- Between-respondent effect		+	+	n.s.	+	n.s.
- Within-respondent effect		n.s.	n.s.	n.s.	n.s.	n.s.

## Conclusion

This study aimed to test to what extent and under which conditions political distrust functions as a push-factor away from the status quo and a conditional pull-factor towards specific alternative decision-making models. To that purpose, we set up a multi-wave panel survey in four European democracies.

We reach three main conclusions. First, political distrust clearly pushes people away from the status quo. High and rising distrust do not merely induce support for political change; it particularly dampens support for the representative model.

Second, political distrust does not indiscriminately pull citizens towards all alternatives. High and rising distrust are rather consistently related to support for direct democracy. Yet, they have mixed effects on expert rule: we find variation across the systematic and dynamic effects of distrust, and variation across countries. Moreover, high and rising distrust do not tend to induce support for authoritarianism (cf. Ouattara & Van der Meer 2023). Predominantly, they tend to push people away from both electoral ánd non-democratic authoritarianism, although we also find evidence that systematic (rather than dynamic) distrust relates to high support for authoritarianism in one country, Sweden.

The third main conclusion reads that personal dispositions matter. We find evidence that the pull of distrust toward direct democracy is significantly stronger among efficacious and populist party leaning citizens. This is in line with recent findings that suggest that the support of populist citizens is principled rather than instrumental, driven by the conviction that including citizens in decision-making enhances the legitimacy of these processes (Werner & Jacobs, 2022). The pull-factor of direct democracy among populist leaning citizens is enhanced by the push away from the status quo that is tied to political distrust. Future research will need to determine what drives this interaction. Although the relationship between populist parties and direct democracy is complex (Gerghina & Pilet 2021a, 2021b), the pull-factor of direct democracy among populist voters may reflect active strategies by populist politicians that do not merely attract voters on that argument, but may also shape these voters (cf. Rooduijn et al., 2016).

Back to distrust. All in all, in line with our model, we find that distrust is an unconditional push factor (away from the status quo) and a conditional pull factor (towards any specific alternative model), based on personal dispositions. These findings underpin the relevance of separating the push and pull factors of high and rising political distrust. This understanding of the effects of political distrust emphasizes their contingency on citizens’ predispositions and regimes’ institutional arrangement.

While political distrust pushes citizens away from the status quo, the nature of the status quo differs across countries. Representative democracy is the status quo in the European parliamentary democracies we studied; levels of authoritarianism and direct democracy tend to be relatively low. This might be one reason why we find an unequivocally negative relationship between political distrust and support for representative democracy. We should extend the systematic test to countries that are characterized as presidential systems (such as the United States), countries with strong direct democratic elements (such as Switzerland), electoral autocracies (such as Hungary or India), or countries with a democratic breakdown (such Nicaragua or Tunesia). Because the status quo is different in these countries, one would expect the relationships between distrust and support for various decision-making models to differ as well. The attractiveness of rivaling models is different, to the extent that they are already integrated into the status quo.

A second contingency may be the temporal context (see Appendix G for an extended discussion). This study took place in late 2020 and early 2021, i.e., after the first wave of the COVID19 pandemic. On the one hand, the context of COVID19 raised support for unelected experts (Lavezzolo et al., 2022). But it is not evident how this context might affect the relationship between political trust and support for rule by unelected experts. To the extent that experts were central to political decisions to combat the pandemic (e.g., lockdown measures), one may expect that distrusters would be less likely to support expert rule, as they were part of the status quo. On the other hand, there is evidence that the COVID19 pandemic raised support for authoritarianism, particularly among trusters (Hirsch, 2022). Yet, again, it is not evident how this might explain why we find that distrust negatively relates to support for authoritarianism in Portugal and the UK, not at all in the Netherlands, but positively in Sweden.

The identification of effects is an ongoing challenge in the political trust literature. While, ultimately, this study is unable to rule out endogeneity, we took two steps to get closer to this elusive objective than previous studies were able to. Theoretically, we formulated stricter direct and conditional hypotheses across a range of outcomes and moderators. We found that distrusters become more likely to support change, to have lower support in the model of the status quo (representative democracy) and to have higher support in direct democracy. These effects are conditional upon individual dispositions, in line with theoretical expectations. It is theoretically unlikely that reverse causality can explain this full range of findings. Moreover, methodologically, we reduced the risk of endogeneity by estimating REWB models on cross-national panel data in a literature dominated by studies in one country at one moment in time. This allowed us to distinguish between the effects of systematic and rising distrust. We found consistent effects for the push-factor (for change, against the status quo), rather consistent effects for the model of direct democracy, and differential effects for expert rule (a positive effect of systematic distrust, and a negative effect of rising distrust). The moderators predominantly have significant effects at the between-person level, possibly because the conditionality is theoretically best understood as a dispositional rather than a fluid position. While this study cannot offer ultimate proof of causality, the theoretical expectations and methodological models offered a stricter test.

All in all, the logical implication of our theoretical model reads that the effects of political distrust on support for rivaling decision-making models are contingent. Political distrust is best understood as an unequivocal push-factor away from the status quo, but not as a straightforward pull-factor towards any rivaling model. Our study only finds rather consistent effects that distrust stimulates support for direct democracy. The dominant pattern suggest the prevalence of dissatisfied democrats: people who lose trust in politics predominantly seem to be attracted to Jane Addams’ (1902) old adage that ‘the cure for the ills of democracy is more democracy’.

## Electronic Supplementary Material

Below is the link to the electronic supplementary material.

Supplementary Material 1

## Data Availability

Data were collected by Kantar, who ensured active consent to participate and publish before every round of data collection. An anonymized replication package (data and code) for this article is deposited at https://osf.io/h5m6t/?view_only=b563c6c47d034d9a903e1daf5b3f3ee1.

## Footnotes

1 Coffé and Michels (2014): representative, direct, and stealth democracy (which incorporates technocracy). Font et al. (2015): representative, participative, and expert rule. Bertsou & Caramani (2022): party-democratic, populist, and technocratic models. Ouattara and Van der Meer (2023): representative democracy, direct democracy, and authoritarianism.

2 This expectation may be contingent on the government status of the populist parties. However, in this study no populist parties were in power.

3 An anonymized replication package for this article is deposited at: https://osf.io/h5m6t/?view_only=b563c6c47d034d9a903e1daf5b3f3ee1.

4 It is possible that some respondents felt conflicted in the choice between options 1 and 2. We therefore ran a robustness check where we dichotomized the measure to distinguish between change (options 1 and 2) and no change (option 3). Results were substantially similar to the main analysis and do not change our conclusions (see Appendix F).

5 Support for representative correlates negatively or non-significantly with support for the rivaling models. Support for the rivaling models (direct democracy, technocracy, authoritarianism) are positively correlated.

6 Straightlining is the tendency to report the same answer category across a series of questions.

7 Unfortunately, we lack both the theory on potential time lags and the sufficiently close time-varying data to test how long it takes for decision-making model support to change once political trust rises or declines. This could take as little as a fraction of a second or as much as several weeks. Hence, we cannot introduce any theoretically relevant time lag to our model.

8 The distinction between the two measures – authoritarian politics and outright undemocratic dictatorships – leads to different effect sizes. However, in Sweden, systematic distrust stimulates both modes of authoritarianism (though authoritarian politics more strongly than dictatorship), whereas in Portugal distrust only significantly undermines support for dictatorship. We can consider two potential partial explanations. On the one hand, group composition may differ in high-trust country Sweden and low-trust country Portugal. On the other hand, the history of Portugal as a military dictatorship until the 1970s may affect political support to this very day.

9 We only find a significant negative interaction effect in the United Kingdom, in line with the theorized mechanism that high efficacy drives distrusters even further away from authoritarian rule (in which they would have less influence).

10 Additional analyses confirm that this interaction effect is indeed driven by populism and not indicative of a nonlinear effect of distrust.
