# Supplementary material for: Pushing and Pulling: The Static and Dynamic Effects of Political Distrust on Support for Representative Democracy and its Rivals
Source: Polit Behav. 2025 Jan 10;47(3):1389–411. doi: 10.1007/s11109-024-09994-y (PMC12396991; doi:10.1007/s11109-024-09994-y)
Supplement: Supplementary file 1 — Supplementary Material 1 [file 11109_2024_9994_MOESM1_ESM.docx]

**Supplementary Materials**

*Pushing and pulling: The static and dynamic effects of political distrust on support for representative democracy and its rivals*

A – Modeling the dependent variables separately………………………………………… 2

B – Results by country……………………………………………………………………. 5

C – Visualizations of conditional effects…………………………………………………. 19

D – Number of participants by country…………………………………………………... 24

E – Control for supporting government parties…………………………………………… 25

F – Modeling the effects on support for political change as a dichotomy………………… 29

G – COVID-19…………………………………………………………………………….. 30

**An anonymized replication package for the article can be found on:** [**https://osf.io/h5m6t/?view_only=b563c6c47d034d9a903e1daf5b3f3ee1**](https://osf.io/h5m6t/?view_only=b563c6c47d034d9a903e1daf5b3f3ee1)

**A – Modeling the dependent variables separately**

**Table A1.** Support for direct democracy and authoritarianism by separate items

|  | Direct democracy 1 | Direct democracy 2 | Authoritarianism 1 | Authoritarianism 2 |
| --- | --- | --- | --- | --- |
| *Within* |  |  |  |  |
| Political distrust | 0.104^***^ | 0.091^***^ | -0.130^***^ | -0.099^***^ |
|  | (0.014) | (0.012) | (0.014) | (0.011) |
| Importance of democracy | -0.017 | -0.029^**^ | -0.095^***^ | -0.127^***^ |
|  | (0.011) | (0.010) | (0.011) | (0.009) |
| *Between* |  |  |  |  |
| Political distrust | 0.417^***^ | 0.443^***^ | 0.002 | -0.035^**^ |
|  | (0.015) | (0.016) | (0.014) | (0.012) |
| Importance of democracy | -0.090^***^ | -0.092^***^ | -0.371^***^ | -0.381 |
|  | (0.014) | (0.015) | (0.014) | (0.011) |
| Female | 0.020 | 0.065^**^ | 0.074^***^ | 0.006 |
|  | (0.023) | (0.024) | (0.022) | (0.017) |
| Age | -0.004^***^ | -0.004^***^ | 0.001 | -0.002^***^ |
|  | (0.001) | (0.001) | (0.001) | (0.001) |
| Education level | -0.049^***^ | -0.092^***^ | -0.099^***^ | -0.040^***^ |
|  | (0.013) | (0.013) | (0.012) | (0.010) |
| Country (ref: UK) |  |  |  |  |
| NL | -0.218^***^ | 0.099^**^ | -0.967^***^ | -0.219^***^ |
|  | (0.031) | (0.032) | (0.029) | (0.023) |
| SE | -0.009 | 0.137^***^ | -0.882^***^ | -0.397^***^ |
|  | (0.032) | (0.033) | (0.030) | (0.024) |
| PT | 0.286^***^ | 0.502^***^ | -0.749^***^ | -0.009 |
|  | (0.035) | (0.037) | (0.033) | (0.027) |
| *Constant* | *2.525^***^* | *2.309^***^* | *5.153^***^* | 4.493^***^ |
|  | *(0.115)* | *(0.121)* | *(0.110)* | (0.088) |
| L2 variation | 0.499^***^ | 0.621^***^ | 0.434^***^ | 0.265^***^ |
|  | (0.014) | (0.015) | (0.013) | (0.008) |
| L1 variation | 0.665^***^ | 0.560^***^ | 0.679^***^ | 0.476^***^ |
|  | (0.009) | (0.008) | (0.009) | (0.007) |
| Observations | 16503 | 16655 | 16750 | 16694 |

Standard errors in parentheses

^+^ *p* < 0.10, ^*^ *p* < 0.05, ^**^ *p* < 0.01, ^***^ *p* < 0.001

Controls for gender, age, level of education, and country

**Table A2.** Support for direct democracy and authoritarianism by separate items, conditional effects of political efficacy

|  | Direct democracy 1 | Direct democracy 2 | Authoritarianism 1 | Authoritarianism 2 |
| --- | --- | --- | --- | --- |
| *Within* |  |  |  |  |
| Political distrust | 0.106^***^ | 0.093^***^ | -0.132^***^ | -0.097^***^ |
|  | (0.014) | (0.012) | (0.014) | (0.011) |
| Importance of democracy | -0.021^+^ | -0.031^**^ | -0.091^***^ | -0.126^***^ |
|  | (0.011) | (0.010) | (0.011) | (0.009) |
| Internal efficacy | 0.055^***^ | 0.035^*^ | -0.042^**^ | 0.013 |
|  | (0.015) | (0.013) | (0.015) | (0.012) |
| *Between* |  |  |  |  |
| Political distrust | 0.406^***^ | 0.431^***^ | -0.006 | -0.032^**^ |
|  | (0.015) | (0.016) | (0.015) | (0.012) |
| Importance of democracy | -0.095^***^ | -0.089^***^ | -0.353^***^ | -0.365^***^ |
|  | (0.014) | (0.015) | (0.014) | (0.011) |
| Internal efficacy | -0.278^***^ | -0.268^***^ | -0.231^***^ | -0.015 |
|  | (0.054) | (0.056) | (0.051) | (0.041) |
| *Interaction* |  |  |  |  |
| Political distrust (within)  * Internal efficacy (within) | -0.007 | -0.032 | -0.057^+^ | -0.061^*^ |
|  | (0.032) | (0.031) | (0.031) | (0.026) |
| Political distrust (between)  * Internal efficacy (between) | 0.097^***^ | 0.077^***^ | 0.037^*^ | -0.022^+^ |
|  | (0.017) | (0.018) | (0.017) | (0.013) |
| *Constant* | *2.590^***^* | *2.334^***^* | *5.082^***^* | *4.382^***^* |
|  | *(0.116)* | *(0.122)* | *(0.111)* | *(0.089)* |
| L2 variation | 0.495^***^ | 0.618^***^ | 0.425^***^ | 0.260^***^ |
|  | (0.014) | (0.015) | (0.013) | (0.008) |
| L1 variation | 0.665^***^ | 0.559^***^ | 0.678^***^ | 0.474^***^ |
|  | (0.009) | (0.008) | (0.009) | (0.007) |
| Observations | 16458 | 16602 | 16701 | 16647 |

Standard errors in parentheses

^+^ *p* < 0.10, ^*^ *p* < 0.05, ^**^ *p* < 0.01, ^***^ *p* < 0.001

Controls for gender, age, level of education, and country

**Table A3.** Support for direct democracy and authoritarianism by separate items, conditional effects of populist party leaning

|  | Direct democracy 1 | Direct democracy 2 | Authoritarianism 1 | Authoritarianism 2 |
| --- | --- | --- | --- | --- |
| *Within* |  |  |  |  |
| Political distrust | 0.056 | 0.081 | -0.095^+^ | -0.008 |
|  | (0.053) | (0.050) | (0.053) | (0.046) |
| Importance of democracy | -0.019 | -0.047^***^ | -0.100^***^ | -0.123^***^ |
|  | (0.012) | (0.011) | (0.012) | (0.010) |
| *Between* |  |  |  |  |
| Political distrust | 0.282^***^ | 0.245^***^ | 0.036 | 0.034 |
|  | (0.042) | (0.044) | (0.041) | (0.032) |
| Importance of democracy | -0.110^***^ | -0.110^***^ | -0.395^***^ | -0.406^***^ |
|  | (0.016) | (0.017) | (0.016) | (0.013) |
| Pop. party leaning | 0.041 | 0.049^+^ | 0.017 | 0.043^*^ |
|  | (0.027) | (0.028) | (0.026) | (0.021) |
| *Interaction* |  |  |  |  |
| Political distrust (within)  * Pop. party leaning (between) | 0.013 | 0.005 | -0.013 | -0.019^+^ |
|  | (0.011) | (0.011) | (0.011) | (0.010) |
| Political distrust (between)  * Pop. Party leaning (between) | 0.015^+^ | 0.022^*^ | -0.007 | -0.014^*^ |
|  | (0.008) | (0.009) | (0.008) | (0.007) |
| *Constant* | *2.614^***^* | *2.443^***^* | *5.187^***^* | *4.426^***^* |
|  | *(0.174)* | *(0.182)* | *(0.171)* | *(0.135)* |
| L2 variation | 0.288^***^ | 0.325^***^ | 0.309^***^ | 0.267^***^ |
|  | (0.026) | (0.026) | (0.030) | (0.021) |
| L1 variation | 0.505^***^ | 0.619^***^ | 0.477^***^ | 0.291^***^ |
|  | (0.015) | (0.016) | (0.014) | (0.009) |
| Random slope  political distrust (within) | 0.583^***^ | 0.470^***^ | 0.601^***^ | 0.396^***^ |
|  | (0.010) | (0.008) | (0.011) | (0.007) |
| Observations | 13990 | 14123 | 14189 | 14151 |

Standard errors in parentheses

^+^ *p* < 0.10, ^*^ *p* < 0.05, ^**^ *p* < 0.01, ^***^ *p* < 0.001

Controls for gender, age, level of education, and country

**B – Results by country**

**Table B1.** Results NL, support for political change by political distrust

|  | Change of leadership | Change of process |
| --- | --- | --- |
| *Within* |  |  |
| Political distrust | 0.449^***^ | 0.446^***^ |
|  | (0.094) | (0.101) |
| Importance of democracy | -0.119 | -0.131 |
|  | (0.094) | (0.097) |
| *Between* |  |  |
| Political distrust | 2.418^***^ | 2.853^***^ |
|  | (0.144) | (0.146) |
| Importance of democracy | -0.552^***^ | -0.584^***^ |
|  | (0.111) | (0.113) |
| *Constant* | *-4.572^***^* | *-6.177^***^* |
|  | *(0.837)* | *(0.848)* |

Unstandardized b-values; Standard errors in parentheses

+ p < 0.10, * p < 0.05, ** p < 0.01, *** p < 0.001

N=4,811

Controls for gender, age, and level of education

**Table B2.** Results UK, support for political change by political distrust

|  | Change of leadership | Change of process |
| --- | --- | --- |
| *Within* |  |  |
| Political distrust | 1.231^***^ | 1.623^***^ |
|  | (0.082) | (0.088) |
| Importance of democracy | -0.351^***^ | -0.376^***^ |
|  | (0.062) | (0.065) |
| *Between* |  |  |
| Political distrust | 1.306^***^ | 1.713^***^ |
|  | (0.093) | (0.098) |
| Importance of democracy | -0.250^**^ | -0.278^***^ |
|  | (0.077) | (0.079) |
| *Constant* | *0.124* | *-1.530^*^* |
|  | *(0.578)* | *(0.605)* |

Unstandardized b-values; Standard errors in parentheses

+ p < 0.10, * p < 0.05, ** p < 0.01, *** p < 0.001

N=4,851

Controls for gender, age, and level of education

**Table B3.** Results SE, support for political change by political distrust

|  | Change of leadership | Change of process |
| --- | --- | --- |
| *Within* |  |  |
| Political distrust | 0.664^***^ | 1.030^***^ |
|  | (0.185) | (0.259) |
| Importance of democracy | 0.127 | 0.027 |
|  | (0.142) | (0.165) |
| *Between* |  |  |
| Political distrust | 3.224^***^ | 4.407^***^ |
|  | (0.178) | (0.194) |
| Importance of democracy | -0.557^***^ | -0.823^***^ |
|  | (0.137) | (0.143) |
| *Constant* | *-3.450^**^* | *-6.980^***^* |
|  | *(1.054)* | *(1.128)* |

Unstandardized b-values; Standard errors in parentheses

+ p < 0.10, * p < 0.05, ** p < 0.01, *** p < 0.001

N=4,350

Controls for gender, age, and level of education

**Table B4.** Results PT, support for political change by political distrust

|  | Change of leadership | Change of process |
| --- | --- | --- |
| *Within* |  |  |
| Political distrust | 0.599^**^ | 0.826^***^ |
|  | (0.189) | (0.202) |
| Importance of democracy | -0.012 | -0.151 |
|  | (0.131) | (0.134) |
| *Between* |  |  |
| Political distrust | 1.814^***^ | 2.439^***^ |
|  | (0.180) | (0.183) |
| Importance of democracy | -0.612^***^ | -0.815^***^ |
|  | (0.149) | (0.151) |
| *Constant* | *1.237* | *-1.068* |
|  | *(1.103)* | *(1.118)* |

Unstandardized b-values; Standard errors in parentheses

+ p < 0.10, * p < 0.05, ** p < 0.01, *** p < 0.001

N=3,052

Controls for gender, age, and level of education

**Table B5.** Results NL, explaining support for decision-making processes by political distrust

|  |  |  |  |  |  |
| --- | --- | --- | --- | --- | --- |
|  | Delegate by electing parliament | Elected professional politicians | Direct democracy | Non-elected expert rule | Authoritarian |
| *Within* |  |  |  |  |  |
| Political distrust | -0.056^**^ | -0.097^***^ | 0.005 | -0.016 | -0.032^*^ |
|  | (0.020) | (0.019) | (0.014) | (0.020) | (0.014) |
| Importance of democracy | 0.026 | 0.066^***^ | 0.005 | -0.007 | -0.003 |
|  | (0.021) | (0.020) | (0.014) | (0.022) | (0.014) |
| *Between* |  |  |  |  |  |
| Political distrust | 0.029 | -0.406^***^ | 0.425^***^ | 0.026 | -0.008 |
|  | (0.022) | (0.020) | (0.025) | (0.022) | (0.018) |
| Importance of democracy | 0.056^*^ | 0.043^*^ | -0.132^***^ | -0.239^***^ | -0.390^***^ |
|  | (0.023) | (0.021) | (0.026) | (0.023) | (0.019) |
| *Constant* | *3.129^***^* | *4.469^***^* | *2.502^***^* | *3.810^***^* | *4.121^***^* |
|  | *(0.175)* | *(0.160)* | *(0.198)* | *(0.174)* | *(0.141)* |
| L2 variation | 0.295^***^ | 0.235^***^ | 0.551^***^ | 0.284^***^ | 0.233^***^ |
|  | (0.018) | (0.015) | (0.022) | (0.018) | (0.012) |
| L1 variation | 0.532^***^ | 0.497^***^ | 0.258^***^ | 0.561^***^ | 0.263^***^ |
|  | (0.014) | (0.013) | (0.007) | (0.015) | (0.007) |
| Observations | 4550 | 4708 | 4744 | 4612 | 4764 |

Unstandardized b-values; Standard errors in parentheses

+ p < 0.10, * p < 0.05, ** p < 0.01, *** p < 0.001

Controls for gender, age, and level of education

**Table B6.** Results UK, explaining support for decision-making processes by political distrust

|  |  |  |  |  |  |
| --- | --- | --- | --- | --- | --- |
|  | Delegate by electing parliament | Elected professional politicians | Direct democracy | Non-elected expert rule | Authoritarian |
| *Within* |  |  |  |  |  |
| Political distrust | -0.225^***^ | -0.489^***^ | 0.194^***^ | -0.142^***^ | -0.200^***^ |
|  | (0.018) | (0.022) | (0.022) | (0.025) | (0.020) |
| Importance of democracy | 0.113^***^ | -0.009 | -0.055^**^ | -0.200^***^ | -0.224^***^ |
|  | (0.014) | (0.017) | (0.017) | (0.019) | (0.016) |
| *Between* |  |  |  |  |  |
| Political distrust | -0.243^***^ | -0.558^***^ | 0.181^***^ | -0.168^***^ | -0.246^***^ |
|  | (0.019) | (0.024) | (0.028) | (0.027) | (0.025) |
| Importance of democracy | 0.183^***^ | -0.025 | -0.042^+^ | -0.208^***^ | -0.274^***^ |
|  | (0.016) | (0.021) | (0.025) | (0.024) | (0.022) |
| *Constant* | *3.577^***^* | *5.225^***^* | *3.153^***^* | *4.814^***^* | *5.291^***^* |
|  | *(0.126)* | *(0.164)* | *(0.192)* | *(0.186)* | *(0.169)* |
| L2 variation | 0.054^***^ | 0.126^***^ | 0.277^***^ | 0.169^***^ | 0.193^***^ |
|  | (0.010) | (0.016) | (0.021) | (0.020) | (0.017) |
| L1 variation | 0.517^***^ | 0.793^***^ | 0.813^***^ | 0.982 | 0.690^***^ |
|  | (0.013) | (0.021) | (0.021) | (0.025) | (0.018) |
| Observations | 4753 | 4737 | 4785 | 4686 | 4810 |

Unstandardized b-values; Standard errors in parentheses

+ p < 0.10, * p < 0.05, ** p < 0.01, *** p < 0.001

Controls for gender, age, and level of education

**Table B7.** Results SE, explaining support for decision-making processes by political distrust

|  |  |  |  |  |  |
| --- | --- | --- | --- | --- | --- |
|  | Delegate by electing parliament | Elected professional politicians | Direct democracy | Non-elected expert rule | Authoritarian |
| *Within* |  |  |  |  |  |
| Political distrust | -0.106^**^ | -0.135^**^ | 0.050^+^ | -0.066 | -0.048^+^ |
|  | (0.039) | (0.044) | (0.030) | (0.041) | (0.028) |
| Importance of democracy | 0.048^+^ | 0.055^+^ | 0.020 | -0.057^*^ | 0.001 |
|  | (0.026) | (0.029) | (0.020) | (0.027) | (0.018) |
| *Between* |  |  |  |  |  |
| Political distrust | -0.250^***^ | -0.467^***^ | 0.622^***^ | 0.188^***^ | 0.157^***^ |
|  | (0.023) | (0.025) | (0.029) | (0.029) | (0.021) |
| Importance of democracy | 0.186^***^ | 0.046^+^ | -0.064^*^ | -0.161^***^ | -0.376^***^ |
|  | (0.024) | (0.025) | (0.028) | (0.028) | (0.021) |
| *Constant* | *3.187^***^* | *4.252^***^* | *1.947^***^* | *4.049^***^* | *3.637^***^* |
|  | *(0.190)* | *(0.202)* | *(0.225)* | *(0.229)* | *(0.166)* |
| L2 variation | 0.292^***^ | 0.332^***^ | 0.590^***^ | 0.518^***^ | 0.286^***^ |
|  | (0.017) | (0.020) | (0.025) | (0.025) | (0.014) |
| L1 variation | 0.421^***^ | 0.540^***^ | 0.260^***^ | 0.469^***^ | 0.228^***^ |
|  | (0.012) | (0.015) | (0.007) | (0.013) | (0.006) |
| Observations | 4115 | 4237 | 4298 | 4256 | 4310 |

Unstandardized b-values; Standard errors in parentheses

+ p < 0.10, * p < 0.05, ** p < 0.01, *** p < 0.001

Controls for gender, age, and level of education

**Table B8.** Results PT, explaining support for decision-making processes by political distrust

|  |  |  |  |  |  |
| --- | --- | --- | --- | --- | --- |
|  | Delegate by electing parliament | Elected professional politicians | Direct democracy | Non-elected expert rule | Authoritarian |
| *Within* |  |  |  |  |  |
| Political distrust | -0.114^**^ | -0.195^***^ | 0.043 | -0.043 | -0.143^***^ |
|  | (0.040) | (0.041) | (0.030) | (0.046) | (0.029) |
| Importance of democracy | 0.053^*^ | -0.006 | 0.003 | 0.018 | -0.021 |
|  | (0.023) | (0.024) | (0.017) | (0.027) | (0.017) |
| *Between* |  |  |  |  |  |
| Political distrust | -0.135^***^ | -0.378^***^ | 0.345^***^ | 0.033 | -0.079^**^ |
|  | (0.025) | (0.033) | (0.033) | (0.035) | (0.030) |
| Importance of democracy | 0.101^***^ | -0.066^*^ | -0.045^+^ | -0.195^***^ | -0.399^***^ |
|  | (0.021) | (0.027) | (0.027) | (0.029) | (0.025) |
| *Constant* | *3.362^***^* | *4.840^***^* | *2.653^***^* | *3.860^***^* | *4.859^***^* |
|  | *(0.171)* | *(0.221)* | *(0.218)* | *(0.236)* | *(0.202)* |
| L2 variation | 0.181^***^ | 0.440^***^ | 0.543^***^ | 0.470^***^ | 0.449^***^ |
|  | (0.018) | (0.030) | (0.029) | (0.034) | (0.024) |
| L1 variation | 0.581^***^ | 0.623^***^ | 0.331^***^ | 0.780^***^ | 0.325^***^ |
|  | (0.019) | (0.020) | (0.011) | (0.025) | (0.010) |
| Observations | 2976 | 3003 | 3025 | 2966 | 3038 |

Unstandardized b-values; Standard errors in parentheses

+ p < 0.10, * p < 0.05, ** p < 0.01, *** p < 0.001

Controls for gender, age, and level of education

**Table B9.** Results NL, conditional effects of internal efficacy

|  | Delegate by electing parliament | Elected professional politicians | Direct  democracy | Non-elected expert rule | Authoritarian |
| --- | --- | --- | --- | --- | --- |
| *Within* |  |  |  |  |  |
| Political distrust | -0.054^**^ | -0.095^***^ | 0.005 | -0.018 | -0.034^*^ |
|  | (0.020) | (0.019) | (0.014) | (0.020) | (0.014) |
| Importance of democracy | 0.024 | 0.065^**^ | 0.004 | -0.004 | -0.001 |
|  | (0.021) | (0.020) | (0.014) | (0.022) | (0.014) |
| Internal efficacy | 0.057^+^ | 0.006 | 0.000 | -0.007 | -0.009 |
|  | (0.029) | (0.028) | (0.020) | (0.030) | (0.020) |
| *Between* |  |  |  |  |  |
| Political distrust | 0.018 | -0.414^***^ | 0.442^***^ | 0.022 | -0.004 |
|  | (0.022) | (0.021) | (0.026) | (0.022) | (0.018) |
| Importance of democracy | 0.044^+^ | 0.050^*^ | -0.139^***^ | -0.231^***^ | -0.381^***^ |
|  | (0.023) | (0.021) | (0.026) | (0.023) | (0.019) |
| Internal efficacy | 0.344^***^ | 0.071 | -0.179^*^ | 0.068 | -0.100 |
|  | (0.076) | (0.070) | (0.088) | (0.077) | (0.062) |
| *Interaction* |  |  |  |  |  |
| Political distrust (within) | -0.170^**^ | -0.063 | -0.011 | 0.023 | -0.028 |
| * Internal efficacy (within) | (0.060) | (0.055) | (0.045) | (0.058) | (0.042) |
| Political distrust (between) | -0.069^**^ | -0.042^+^ | 0.078^*^ | -0.038 | 0.009 |
| * Internal efficacy (between) | (0.027) | (0.025) | (0.031) | (0.027) | (0.022) |
| *Constant* | *3.342^***^* | *4.436^***^* | *2.519^***^* | *3.763^***^* | *4.048^***^* |
|  | *(0.175)* | *(0.162)* | *(0.200)* | *(0.177)* | *(0.142)* |
| L2 variation | 0.283^***^ | 0.234^***^ | 0.548^***^ | 0.284^***^ | 0.227^***^ |
|  | (0.017) | (0.015) | (0.022) | (0.018) | (0.011) |
| L1 variation | 0.530^***^ | 0.497^***^ | 0.257^***^ | 0.561^***^ | 0.261^***^ |
|  | (0.014) | (0.013) | (0.007) | (0.015) | (0.007) |
| Observations | 4534 | 4691 | 4726 | 4596 | 4745 |

Standard errors in parentheses

^+^ *p* < 0.10, ^*^ *p* < 0.05, ^**^ *p* < 0.01, ^***^ *p* < 0.001

Controls for gender, age, level of education, and country

**Table B10.** Results UK, conditional effects of internal efficacy

|  | Delegate by electing parliament | Elected professional politicians | Direct  democracy | Non-elected expert rule | Authoritarian |
| --- | --- | --- | --- | --- | --- |
| *Within* |  |  |  |  |  |
| Political distrust | -0.218^***^ | -0.490^***^ | 0.200^***^ | -0.139^***^ | -0.200^***^ |
|  | (0.018) | (0.022) | (0.022) | (0.025) | (0.021) |
| Importance of democracy | 0.103^***^ | 0.004 | -0.065^***^ | -0.197^***^ | -0.216^***^ |
|  | (0.014) | (0.017) | (0.018) | (0.019) | (0.016) |
| Internal efficacy | 0.098^***^ | -0.069^**^ | 0.078^***^ | -0.018 | -0.051^*^ |
|  | (0.019) | (0.023) | (0.024) | (0.026) | (0.022) |
| *Between* |  |  |  |  |  |
| Political distrust | -0.233^***^ | -0.536^***^ | 0.179^***^ | -0.139^***^ | -0.219^***^ |
|  | (0.020) | (0.026) | (0.030) | (0.030) | (0.027) |
| Importance of democracy | 0.167^***^ | 0.007 | -0.057^*^ | -0.202^***^ | -0.259^***^ |
|  | (0.017) | (0.022) | (0.025) | (0.025) | (0.022) |
| Internal efficacy | 0.197^*^ | 0.136 | 0.007 | 0.304^*^ | 0.237^*^ |
|  | (0.088) | (0.115) | (0.135) | (0.131) | (0.118) |
| *Interaction* |  |  |  |  |  |
| Political distrust (within) | -0.003 | -0.065^+^ | -0.015 | -0.049 | -0.064^+^ |
| * Internal efficacy (within) | (0.029) | (0.037) | (0.040) | (0.041) | (0.036) |
| Political distrust (between) | -0.034 | -0.082^*^ | 0.017 | -0.096^*^ | -0.084^*^ |
| * Internal efficacy (between) | (0.027) | (0.035) | (0.041) | (0.040) | (0.036) |
| *Constant* | *3.623^***^* | *4.942^***^* | *3.239^***^* | *4.660^***^* | *5.086^***^* |
|  | *(0.132)* | *(0.172)* | *(0.202)* | *(0.196)* | *(0.177)* |
| L2 variation | 0.053^***^ | 0.119^***^ | 0.274^***^ | 0.165^***^ | 0.188^***^ |
|  | (0.010) | (0.016) | (0.021) | (0.020) | (0.016) |
| L1 variation | 0.511^***^ | 0.790^***^ | 0.813^***^ | 0.983 | 0.690^***^ |
|  | (0.013) | (0.020) | (0.021) | (0.026) | (0.018) |
| Observations | 4733 | 4718 | 4765 | 4667 | 4791 |

Standard errors in parentheses

^+^ *p* < 0.10, ^*^ *p* < 0.05, ^**^ *p* < 0.01, ^***^ *p* < 0.001

Controls for gender, age, level of education, and country

**Table B11.** Results SE, conditional effects of internal efficacy

|  | Delegate by electing parliament | Elected professional politicians | Direct  democracy | Non-elected expert rule | Authoritarian |
| --- | --- | --- | --- | --- | --- |
| *Within* |  |  |  |  |  |
| Political distrust | -0.098^*^ | -0.132^**^ | 0.053^+^ | -0.064 | -0.053^+^ |
|  | (0.039) | (0.044) | (0.030) | (0.041) | (0.028) |
| Importance of democracy | 0.048^+^ | 0.053^+^ | 0.019 | -0.058^*^ | -0.001 |
|  | (0.026) | (0.029) | (0.020) | (0.027) | (0.018) |
| Internal efficacy | 0.031 | 0.040 | 0.048^*^ | 0.071^*^ | 0.068^***^ |
|  | (0.029) | (0.032) | (0.022) | (0.029) | (0.020) |
| *Between* |  |  |  |  |  |
| Political distrust | -0.204^***^ | -0.498^***^ | 0.587^***^ | 0.142^***^ | 0.143^***^ |
|  | (0.025) | (0.027) | (0.031) | (0.031) | (0.022) |
| Importance of democracy | 0.162^***^ | 0.051^*^ | -0.058^*^ | -0.143^***^ | -0.364^***^ |
|  | (0.023) | (0.025) | (0.028) | (0.028) | (0.021) |
| Internal efficacy | 0.435^***^ | -0.284^**^ | -0.332^***^ | -0.438^***^ | -0.165^*^ |
|  | (0.079) | (0.087) | (0.098) | (0.098) | (0.072) |
| *Interaction* |  |  |  |  |  |
| Political distrust (within) | 0.292^*^ | -0.008 | 0.078 | 0.059 | 0.032 |
| * Internal efficacy (within) | (0.124) | (0.138) | (0.107) | (0.135) | (0.095) |
| Political distrust (between) | -0.082^**^ | 0.080^**^ | 0.091^**^ | 0.095^**^ | 0.018 |
| * Internal efficacy (between) | (0.027) | (0.030) | (0.033) | (0.033) | (0.024) |
| *Constant* | *3.175^***^* | *4.317^***^* | *2.008^***^* | *4.078^***^* | *3.603^***^* |
|  | *(0.188)* | *(0.205)* | *(0.228)* | *(0.230)* | *(0.168)* |
| L2 variation | 0.267^***^ | 0.328^***^ | 0.584^***^ | 0.498^***^ | 0.281^***^ |
|  | (0.016) | (0.020) | (0.025) | (0.025) | (0.013) |
| L1 variation | 0.420^***^ | 0.540^***^ | 0.259^***^ | 0.468^***^ | 0.223^***^ |
|  | (0.012) | (0.015) | (0.007) | (0.013) | (0.006) |
| Observations | 4108 | 4228 | 4289 | 4250 | 4301 |

Standard errors in parentheses

^+^ *p* < 0.10, ^*^ *p* < 0.05, ^**^ *p* < 0.01, ^***^ *p* < 0.001

Controls for gender, age, level of education, and country

**Table B12.** Results PT, conditional effects of internal efficacy

|  | Delegate by electing parliament | Elected professional politicians | Direct  democracy | Non-elected expert rule | Authoritarian |
| --- | --- | --- | --- | --- | --- |
| *Within* |  |  |  |  |  |
| Political distrust | -0.102^*^ | -0.195^***^ | 0.047 | -0.041 | -0.140^***^ |
|  | (0.040) | (0.041) | (0.030) | (0.047) | (0.030) |
| Importance of democracy | 0.053^*^ | -0.006 | 0.004 | 0.018 | -0.020 |
|  | (0.023) | (0.024) | (0.017) | (0.027) | (0.017) |
| Internal efficacy | 0.084^*^ | 0.022 | 0.044^+^ | 0.031 | 0.032 |
|  | (0.034) | (0.036) | (0.026) | (0.040) | (0.026) |
| *Between* |  |  |  |  |  |
| Political distrust | -0.128^***^ | -0.402^***^ | 0.318^***^ | 0.028 | -0.090^**^ |
|  | (0.026) | (0.033) | (0.034) | (0.036) | (0.031) |
| Importance of democracy | 0.086^***^ | -0.033 | -0.046^+^ | -0.183^***^ | -0.382^***^ |
|  | (0.021) | (0.027) | (0.027) | (0.030) | (0.025) |
| Internal efficacy | 0.164^+^ | -0.419^***^ | -0.370^**^ | -0.114 | -0.198^+^ |
|  | (0.096) | (0.122) | (0.123) | (0.133) | (0.113) |
| *Interaction* |  |  |  |  |  |
| Political distrust (within) | 0.118 | -0.021 | -0.128 | -0.105 | -0.041 |
| * Internal efficacy (within) | (0.098) | (0.110) | (0.091) | (0.123) | (0.085) |
| Political distrust (between) | -0.016 | 0.055 | 0.119^**^ | 0.009 | 0.022 |
| * Internal efficacy (between) | (0.029) | (0.037) | (0.037) | (0.040) | (0.034) |
| *Constant* | *3.444^***^* | *4.691^***^* | *2.740^***^* | *3.792^***^* | *4.779^***^* |
|  | *(0.173)* | *(0.221)* | *(0.222)* | *(0.240)* | *(0.205)* |
| L2 variation | 0.175^***^ | 0.404^***^ | 0.538^***^ | 0.464^***^ | 0.438^***^ |
|  | (0.018) | (0.028) | (0.028) | (0.034) | (0.024) |
| L1 variation | 0.579^***^ | 0.624^***^ | 0.330^***^ | 0.781^***^ | 0.325^***^ |
|  | (0.019) | (0.020) | (0.011) | (0.026) | (0.011) |
| Observations | 2970 | 2996 | 3019 | 2963 | 3032 |

Standard errors in parentheses

^+^ *p* < 0.10, ^*^ *p* < 0.05, ^**^ *p* < 0.01, ^***^ *p* < 0.001

Controls for gender, age, level of education, and country

**Table B13.** Results NL, conditional effects of populist party leaning

|  | Delegate by electing parliament | Elected professional politicians | Direct  democracy | Non-elected expert rule | Authoritarian |
| --- | --- | --- | --- | --- | --- |
| *Within* |  |  |  |  |  |
| Political distrust | -0.106^*^ | -0.120^**^ | 0.003 | 0.016 | -0.035 |
|  | (0.046) | (0.043) | (0.030) | (0.045) | (0.030) |
| Importance of democracy | 0.020 | 0.065^**^ | 0.004 | -0.004 | -0.001 |
|  | (0.024) | (0.022) | (0.016) | (0.024) | (0.016) |
| *Between* |  |  |  |  |  |
| Political distrust | -0.188^***^ | -0.159^***^ | 0.117^*^ | 0.053 | 0.069^+^ |
|  | (0.049) | (0.044) | (0.054) | (0.049) | (0.040) |
| Importance of democracy | 0.051^*^ | 0.006 | -0.111^***^ | -0.260^***^ | -0.404^***^ |
|  | (0.026) | (0.023) | (0.028) | (0.026) | (0.021) |
| Populist party leaning | -0.048^+^ | 0.056^*^ | 0.029 | 0.043 | 0.023 |
|  | (0.027) | (0.024) | (0.029) | (0.027) | (0.022) |
| *Interaction* |  |  |  |  |  |
| Political distrust (within) | 0.011 | 0.006 | 0.002 | -0.010 | 0.000 |
| * Pop. party leaning (between) | (0.010) | (0.010) | (0.007) | (0.010) | (0.007) |
| Political distrust (between) | 0.032^***^ | -0.035^***^ | 0.029^**^ | -0.010 | -0.012^+^ |
| * Pop. Party leaning (between) | (0.009) | (0.008) | (0.009) | (0.009) | (0.007) |
| *Constant* | *3.511^***^* | *4.324^***^* | *2.570^***^* | *3.749^***^* | *4.029^***^* |
|  | *(0.229)* | *(0.204)* | *(0.249)* | *(0.230)* | *(0.187)* |
| L2 variation | 0.015^***^ | 0.021^***^ | 0.000 | 0.007^*^ | 0.000^***^ |
|  | (0.014) | (0.013) | (0.000) | (0.016) | (0.000) |
| L1 variation | 0.288^***^ | 0.213^***^ | 0.492^***^ | 0.289^***^ | 0.234^***^ |
|  | (0.019) | (0.015) | (0.022) | (0.020) | (0.013) |
| Random slope | 0.545^***^ | 0.491^***^ | 0.259^***^ | 0.565^***^ | 0.270^***^ |
| Political distrust (within) | (0.016) | (0.015) | (0.007) | (0.017) | (0.007) |
| Observations | 3888 | 4002 | 4023 | 3932 | 4037 |

Standard errors in parentheses

^+^ *p* < 0.10, ^*^ *p* < 0.05, ^**^ *p* < 0.01, ^***^ *p* < 0.001

Controls for gender, age, level of education, and country

**Table B14.** Results UK, conditional effects of populist party leaning

|  | Delegate by electing parliament | Elected professional politicians | Direct  democracy | Non-elected expert rule | Authoritarian |
| --- | --- | --- | --- | --- | --- |
| *Within* |  |  |  |  |  |
| Political distrust | -0.495^*^ | -0.480^+^ | 0.701^*^ | 0.319 | 0.263 |
|  | (0.199) | (0.248) | (0.281) | (0.266) | (0.248) |
| Importance of democracy | 0.110^***^ | -0.010 | -0.077^***^ | -0.208^***^ | -0.222^***^ |
|  | (0.014) | (0.018) | (0.018) | (0.020) | (0.017) |
| *Between* |  |  |  |  |  |
| Political distrust | -0.030 | -0.666^**^ | -0.046 | -0.071 | 0.233 |
|  | (0.180) | (0.235) | (0.275) | (0.269) | (0.243) |
| Importance of democracy | 0.185^***^ | -0.018 | -0.061^*^ | -0.204^***^ | -0.275^***^ |
|  | (0.017) | (0.023) | (0.026) | (0.026) | (0.023) |
| Populist party leaning | 0.122 | -0.155 | -0.075 | 0.099 | 0.316^+^ |
|  | (0.127) | (0.167) | (0.196) | (0.191) | (0.173) |
| *Interaction* |  |  |  |  |  |
| Political distrust (within) | 0.056 | 0.001 | -0.104^+^ | -0.096^+^ | -0.097^+^ |
| * Pop. party leaning (between) | (0.042) | (0.052) | (0.059) | (0.056) | (0.052) |
| Political distrust (between) | -0.047 | 0.024 | 0.043 | -0.022 | -0.106^*^ |
| * Pop. party leaning (between) | (0.038) | (0.049) | (0.058) | (0.056) | (0.051) |
| *Constant* | *3.053^***^* | *5.948^***^* | *3.644^***^* | *4.350^***^* | *3.826^***^* |
|  | *(0.621)* | *(0.815)* | *(0.954)* | *(0.932)* | *(0.844)* |
| L2 variation | 0.118^***^ | 0.165^***^ | 0.417^***^ | 0.131^***^ | 0.270^***^ |
|  | (0.020) | (0.033) | (0.048) | (0.034) | (0.037) |
| L1 variation | 0.076^***^ | 0.150^***^ | 0.339^***^ | 0.193^***^ | 0.237^***^ |
|  | (0.010) | (0.017) | (0.022) | (0.021) | (0.017) |
| Random slope | 0.438^***^ | 0.700^***^ | 0.609^***^ | 0.906^**^ | 0.558^***^ |
| Political distrust (within) | (0.014) | (0.023) | (0.021) | (0.029) | (0.019) |
| Observations | 4330 | 4315 | 4360 | 4272 | 4378 |

Standard errors in parentheses

^+^ *p* < 0.10, ^*^ *p* < 0.05, ^**^ *p* < 0.01, ^***^ *p* < 0.001

Controls for gender, age, level of education, and country

**Table B15.** Results SE, conditional effects of populist party leaning

|  | Delegate by electing parliament | Elected professional politicians | Direct  democracy | Non-elected expert rule | Authoritarian |
| --- | --- | --- | --- | --- | --- |
| *Within* |  |  |  |  |  |
| Political distrust | -0.184 | -0.094 | 0.070 | -0.021 | -0.056 |
|  | (0.136) | (0.152) | (0.099) | (0.137) | (0.101) |
| Importance of democracy | 0.063^*^ | 0.046 | 0.031 | -0.076^**^ | 0.011 |
|  | (0.028) | (0.031) | (0.021) | (0.028) | (0.020) |
| *Between* |  |  |  |  |  |
| Political distrust | -0.391^***^ | -0.373^***^ | 0.364^***^ | 0.383^***^ | -0.052 |
|  | (0.076) | (0.083) | (0.093) | (0.095) | (0.069) |
| Importance of democracy | 0.185^***^ | 0.026 | -0.090^**^ | -0.183^***^ | -0.398^***^ |
|  | (0.026) | (0.028) | (0.030) | (0.031) | (0.023) |
| Populist party leaning | -0.088 | -0.008 | -0.039 | 0.117^+^ | -0.148^**^ |
|  | (0.055) | (0.060) | (0.067) | (0.069) | (0.050) |
| *Interaction* |  |  |  |  |  |
| Political distrust (within) | 0.023 | -0.001 | -0.007 | -0.004 | 0.000 |
| * Pop. party leaning (between) | (0.034) | (0.039) | (0.025) | (0.035) | (0.026) |
| Political distrust (between) | 0.036^*^ | -0.012 | 0.049^*^ | -0.043^+^ | 0.054^***^ |
| * Pop. party leaning (between) | (0.018) | (0.019) | (0.022) | (0.022) | (0.016) |
| *Constant* | *3.573^***^* | *4.323^***^* | *2.386^***^* | *3.728^***^* | *4.334^***^* |
|  | *(0.286)* | *(0.310)* | *(0.345)* | *(0.355)* | *(0.257)* |
| L2 variation | 0.206^***^ | 0.286^***^ | 0.072^***^ | 0.187^***^ | 0.196^***^ |
|  | (0.081) | (0.104) | (0.039) | (0.071) | (0.046) |
| L1 variation | 0.276^***^ | 0.325^***^ | 0.562^***^ | 0.517^***^ | 0.285^***^ |
|  | (0.017) | (0.021) | (0.025) | (0.026) | (0.014) |
| Random slope | 0.404^***^ | 0.518^***^ | 0.246^***^ | 0.447^***^ | 0.210^***^ |
| Political distrust (within) | (0.014) | (0.017) | (0.008) | (0.014) | (0.007) |
| Observations | 3710 | 3802 | 3856 | 3828 | 3868 |

Standard errors in parentheses

^+^ *p* < 0.10, ^*^ *p* < 0.05, ^**^ *p* < 0.01, ^***^ *p* < 0.001

Controls for gender, age, level of education, and country

**Table B16.** Results PT, conditional effects of populist party leaning

|  | Delegate by electing parliament | Elected professional politicians | Direct  democracy | Non-elected expert rule | Authoritarian |
| --- | --- | --- | --- | --- | --- |
| *Within* |  |  |  |  |  |
| Political distrust | -0.155 | -0.366^**^ | 0.120 | 0.168 | 0.057 |
|  | (0.123) | (0.121) | (0.095) | (0.142) | (0.095) |
| Importance of democracy | 0.069^*^ | -0.026 | -0.009 | -0.025 | -0.046^*^ |
|  | (0.029) | (0.032) | (0.023) | (0.035) | (0.023) |
| *Between* |  |  |  |  |  |
| Political distrust | -0.224^**^ | -0.337^***^ | 0.195^+^ | 0.095 | -0.095 |
|  | (0.076) | (0.100) | (0.103) | (0.109) | (0.097) |
| Importance of democracy | 0.155^***^ | -0.097^**^ | -0.071^+^ | -0.227^***^ | -0.471^***^ |
|  | (0.028) | (0.037) | (0.038) | (0.041) | (0.036) |
| Populist party leaning | -0.047 | -0.063 | -0.058 | 0.005 | -0.071 |
|  | (0.051) | (0.067) | (0.069) | (0.073) | (0.065) |
| *Interaction* |  |  |  |  |  |
| Political distrust (within) | 0.007 | 0.040 | -0.018 | -0.052^+^ | -0.054^**^ |
| * Pop. party leaning (between) | (0.027) | (0.027) | (0.021) | (0.031) | (0.021) |
| Political distrust (between) | 0.014 | 0.007 | 0.027 | -0.010 | 0.008 |
| * Pop. party leaning (between) | (0.016) | (0.021) | (0.022) | (0.023) | (0.021) |
| *Constant* | *3.355^***^* | *5.157^***^* | *3.217^***^* | *4.062^***^* | *5.638^***^* |
|  | *(0.302)* | *(0.399)* | *(0.408)* | *(0.434)* | *(0.386)* |
| L2 variation | 0.186^***^ | 0.020 | 0.098^***^ | 0.162^**^ | 0.098^***^ |
|  | (0.067) | (0.081) | (0.041) | (0.098) | (0.047) |
| L1 variation | 0.187^***^ | 0.412^***^ | 0.573^***^ | 0.497^***^ | 0.499^***^ |
|  | (0.021) | (0.036) | (0.036) | (0.042) | (0.033) |
| Random slope | 0.507^***^ | 0.642^***^ | 0.311^***^ | 0.738^***^ | 0.316^***^ |
| Political distrust (within) | (0.023) | (0.031) | (0.014) | (0.034) | (0.015) |
| Observations | 2010 | 2020 | 2029 | 2004 | 2038 |

Standard errors in parentheses

^+^ *p* < 0.10, ^*^ *p* < 0.05, ^**^ *p* < 0.01, ^***^ *p* < 0.001

Controls for gender, age, level of education, and country

**C – Visualizations of conditional effects**


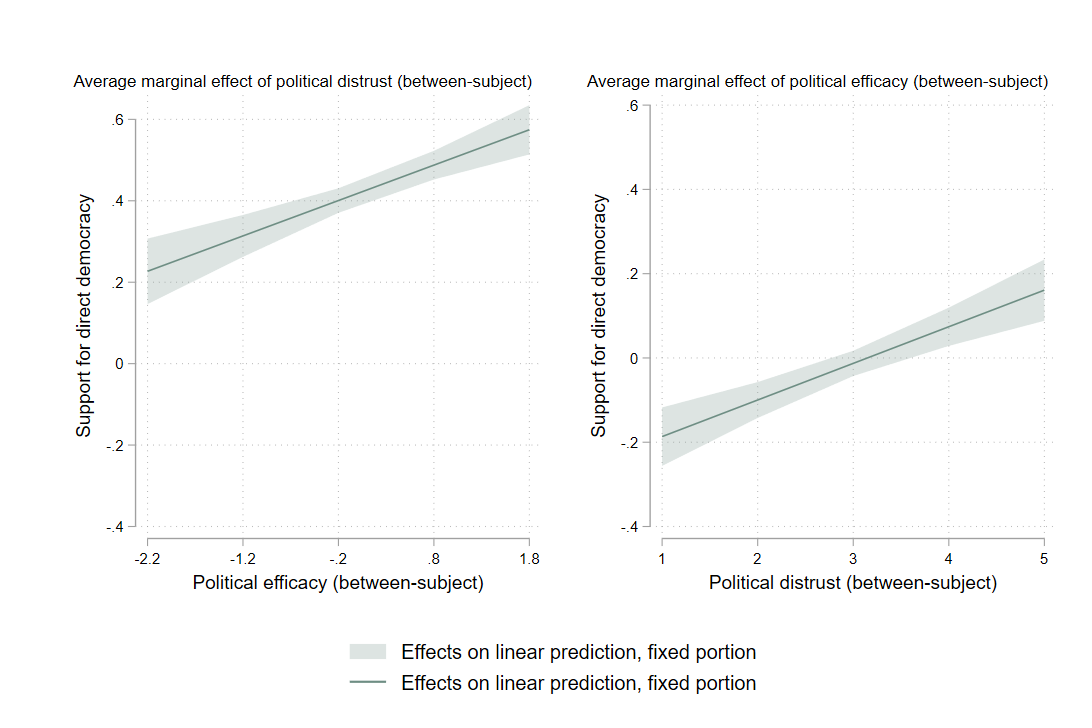
**Figure C1.** The average marginal effect of political distrust (between-subject) by political efficacy and vice versa

**
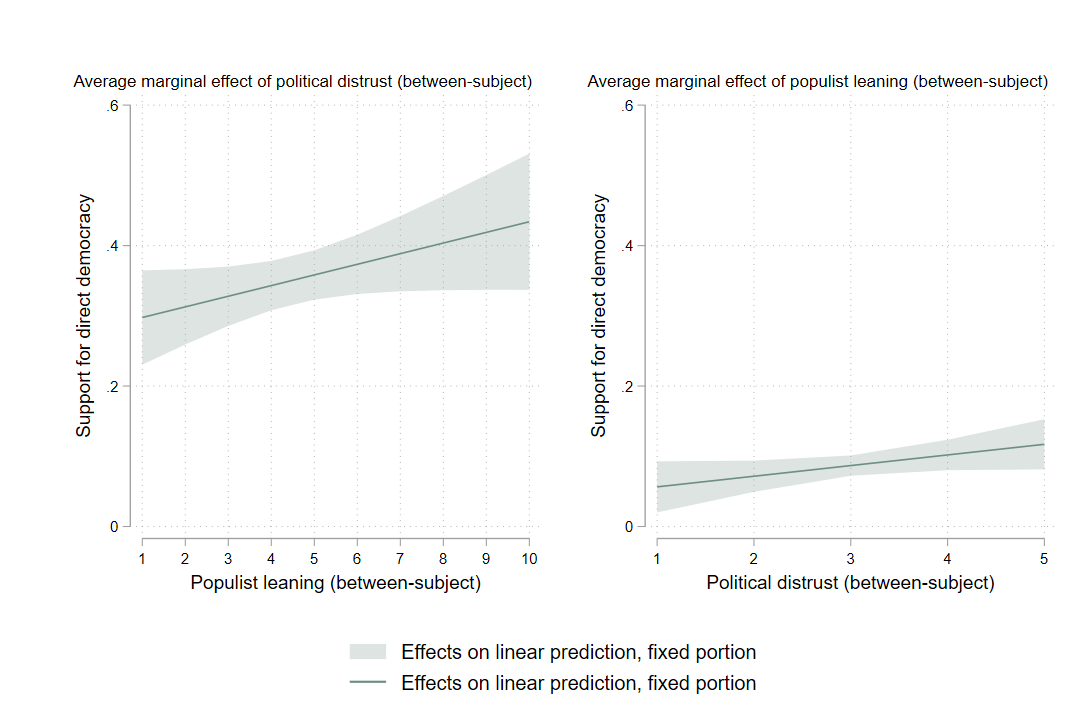
Figure C2.** The average marginal effect of political distrust (between-subject) by populist leaning and vice versa


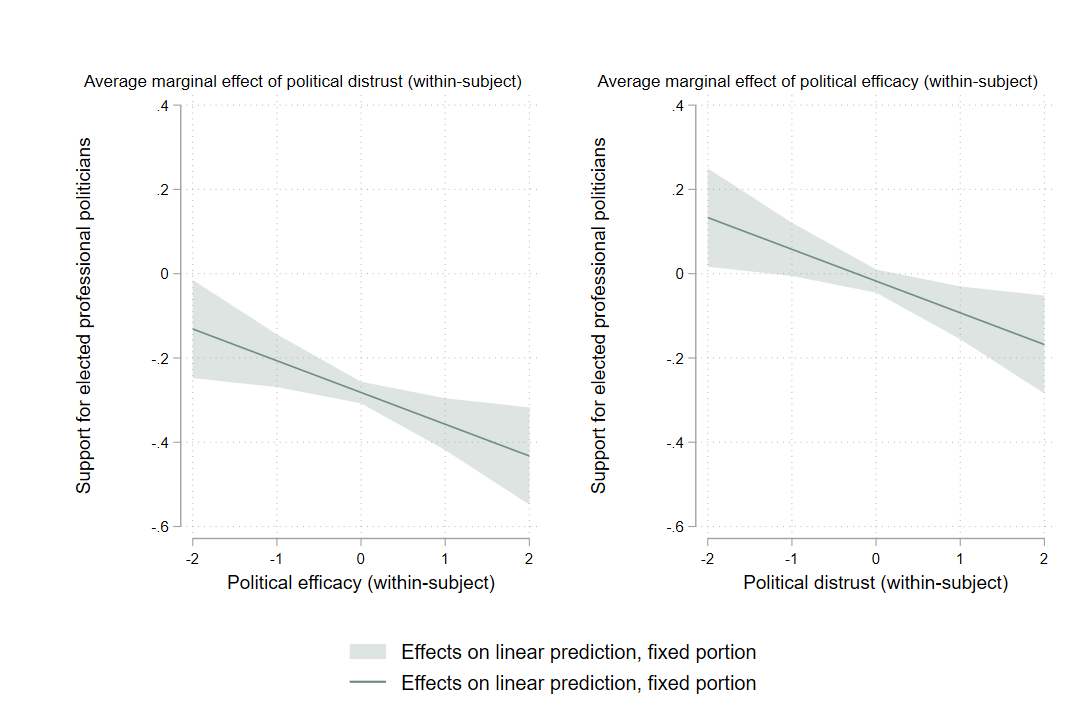
**
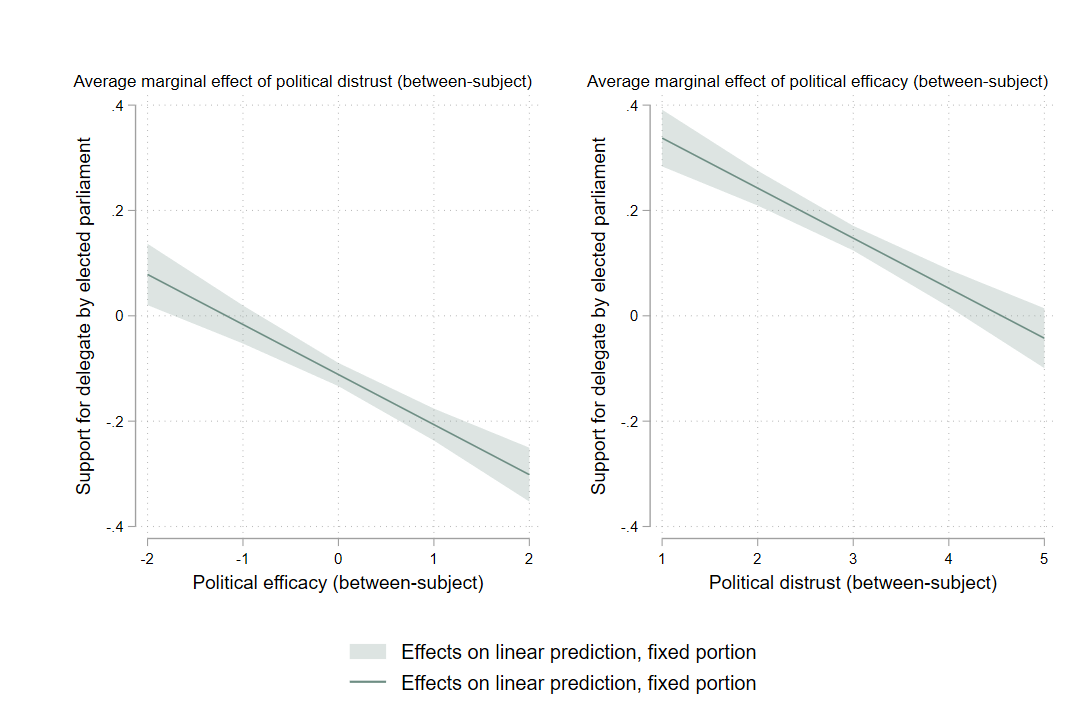
Figure C3.** The effect of political distrust on support for representative democracy, conditional by efficacy


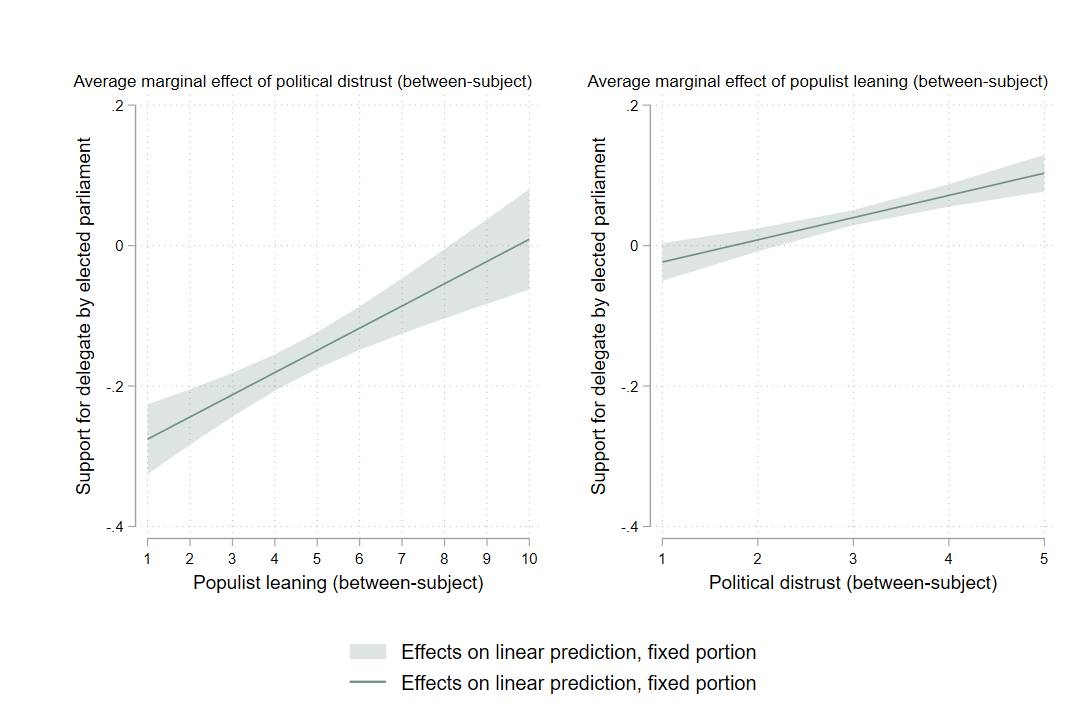
**Figure C4.** The effect of political distrust on support for representative democracy, conditional by populist leaning


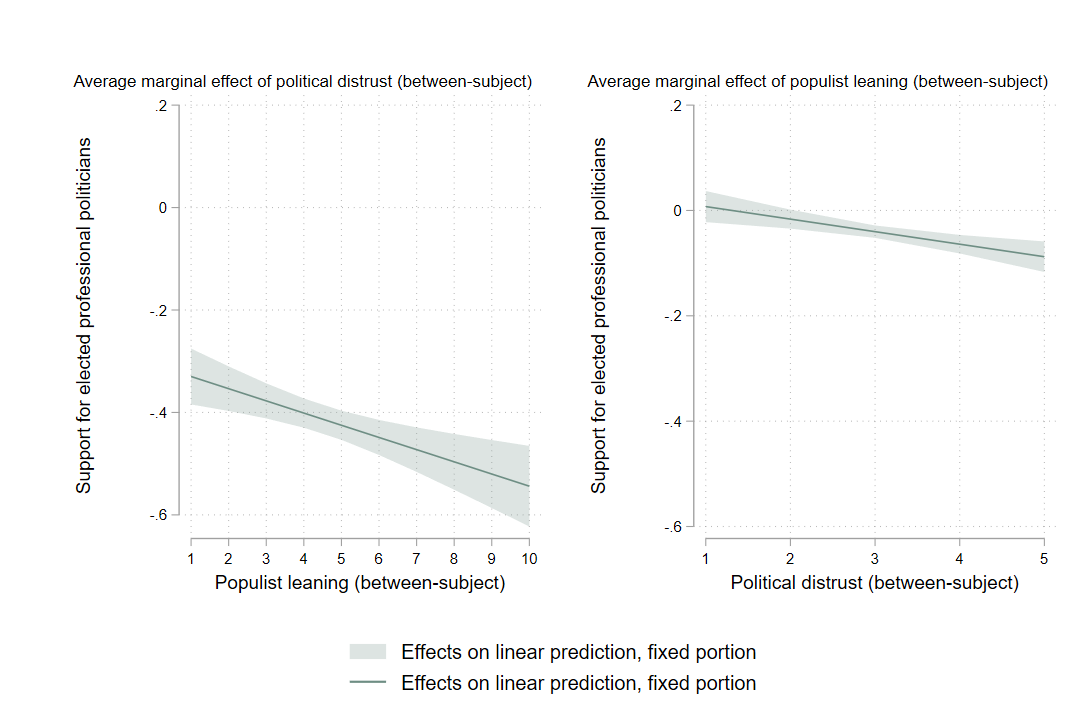


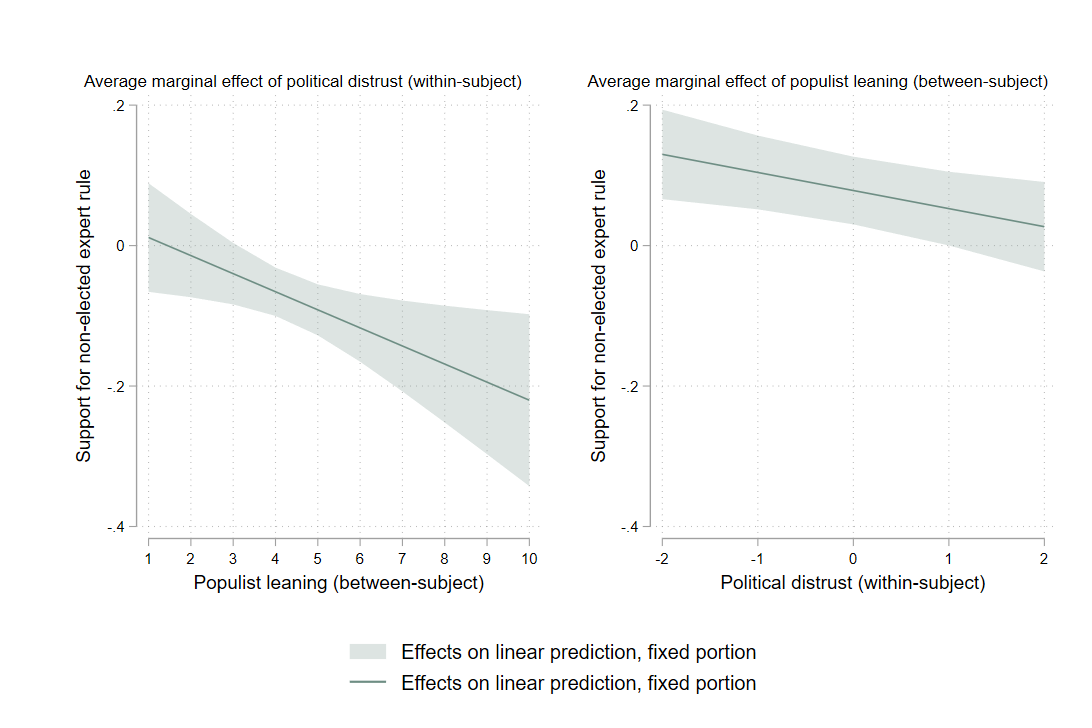

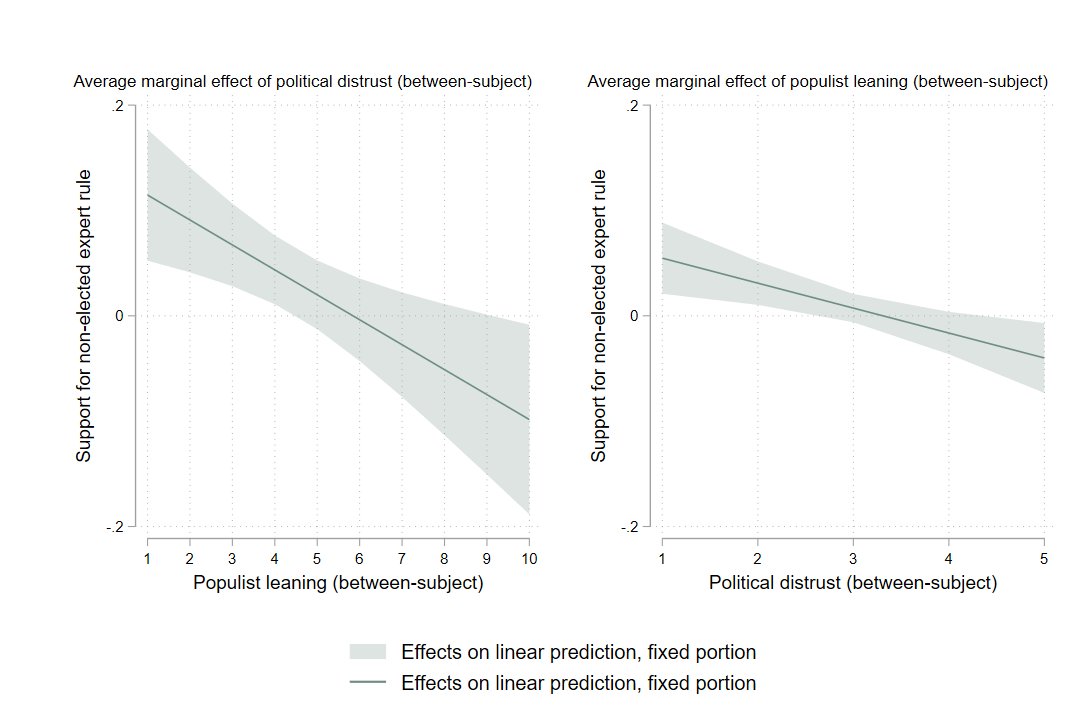
**Figure C5.** The effect of political distrust on support for expert rule, conditional by populist leaning

**D – Number of participants by country**

| **Table D1.** Number of participants per wave per country | | | | | |
| --- | --- | --- | --- | --- | --- |
|  | UK | NL | SE | PT | Total |
| N wave 1 | 2,387 | 2,006 | 2,408 | 1,527 | 8,328 |
| N wave 2 | 1,911 | 1,760 | 1,634 | 1,190 | 6,495 |
| N wave 3 | 1,645 | 1,614 | 1,322 | 901 | 5,482 |
| Total observations | 5,943 | 5,380 | 5,364 | 3,618 | 20,305 |

| **Table D2.** Number of participants per wave per country after excluding straightliners and respondents who only participate in one wave | | | | | |
| --- | --- | --- | --- | --- | --- |
|  | UK | NL | SE | PT | Total |
| N wave 1 | 1,774 | 1,696 | 1,581 | 1,122 | 6,173 |
| N wave 2 | 1,774 | 1,695 | 1,580 | 1,122 | 6,171 |
| N wave 3 | 1,524 | 1,556 | 1,276 | 846 | 5,202 |
| Total observations | 5,072 | 4,947 | 4,437 | 3,090 | 17,546 |

**E – Control for supporting government parties**

**Table E1.** Support for political change by political distrust, controlling for support government parties

|  | Change of leadership | Change of process |
| --- | --- | --- |
| *Within* |  |  |
| Political distrust | 0.900^***^ | 1.173^***^ |
|  | (0.055) | (0.060) |
| Importance of democracy | -0.234^***^ | -0.292^***^ |
|  | (0.046) | (0.048) |
| *Between* |  |  |
| Political distrust | 1.828^***^ | 2.466^***^ |
|  | (0.066) | (0.069) |
| Importance of democracy | -0.437^***^ | -0.583^***^ |
|  | (0.053) | (0.055) |
| *Country (ref: UK)* |  |  |
| NL | -1.961^***^ | -1.641^***^ |
|  | (0.105) | (0.108) |
| SE | -0.128 | -1.096^***^ |
|  | (0.105) | (0.116) |
| PT | 0.748^***^ | 0.823^***^ |
|  | (0.122) | (0.126) |
| *Ref: Support government party* |  |  |
| opposition | 1.519^***^ | 1.274^***^ |
|  | (0.091) | (0.097) |
| don't know/no party | 0.933^***^ | 0.749^***^ |
|  | (0.109) | (0.116) |
| *Constant* | *-1.984^***^* | *-3.811^***^* |
|  | *(0.411)* | *(0.425)* |

Unstandardized b-values; Standard errors in parentheses

^+^ *p* < 0.10, ^*^ *p* < 0.05, ^**^ *p* < 0.01, ^***^ *p* < 0.001

N=17,064

Controls for gender, age, and level of education

**Table E2.** Explaining support for decision-making processes by political distrust, controlling for supporting government parties

|  | Delegate by electing parliament | Elected professional politicians | Direct democracy | Non-elected expert rule | Authoritarian |
| --- | --- | --- | --- | --- | --- |
| *Within* |  |  |  |  |  |
| Political distrust | -0.143^***^ | -0.284^***^ | 0.097^***^ | -0.076^***^ | -0.115^***^ |
|  | (0.012) | (0.013) | (0.011) | (0.014) | (0.010) |
| Importance of democracy | 0.078^***^ | 0.021^+^ | -0.025^**^ | -0.099^***^ | -0.110^***^ |
|  | (0.009) | (0.010) | (0.009) | (0.011) | (0.008) |
| *Between* |  |  |  |  |  |
| Political distrust | -0.133^***^ | -0.430^***^ | 0.401^***^ | 0.030^*^ | 0.005 |
|  | (0.012) | (0.013) | (0.015) | (0.015) | (0.012) |
| Importance of democracy | 0.124^***^ | -0.002 | -0.087^***^ | -0.210^***^ | -0.379^***^ |
|  | (0.011) | (0.012) | (0.013) | (0.013) | (0.011) |
| *Country (ref: UK)* |  |  |  |  |  |
| NL | -0.415^***^ | 0.151^***^ | -0.077^**^ | -0.140^***^ | -0.589^***^ |
|  | (0.023) | (0.025) | (0.029) | (0.028) | (0.023) |
| SE | -0.100^***^ | -0.155^***^ | 0.027 | 0.388^***^ | -0.628^***^ |
|  | (0.024) | (0.026) | (0.030) | (0.030) | (0.025) |
| PT | -0.162^***^ | -0.333^***^ | 0.380^***^ | 0.206^***^ | -0.371^***^ |
|  | (0.026) | (0.028) | (0.033) | (0.032) | (0.027) |
| *Ref: Support government party* | |  |  |  |  |
| opposition | 0.055^**^ | -0.144^***^ | 0.176^***^ | 0.057^*^ | -0.104^***^ |
|  | (0.020) | (0.022) | (0.026) | (0.025) | (0.021) |
| don't know/no party | -0.101^***^ | -0.160^***^ | 0.085^**^ | -0.008 | -0.122^***^ |
|  | (0.025) | (0.027) | (0.031) | (0.031) | (0.026) |
| *Constant* | *3.453^***^* | *4.829^***^* | *2.370^***^* | *3.995^***^* | *4.861^***^* |
|  | *(0.086)* | *(0.094)* | *(0.108)* | *(0.106)* | *(0.088)* |
| L2 variation | 0.216^***^ | 0.267^***^ | 0.509^***^ | 0.366^***^ | 0.295^***^ |
|  | (0.008) | (0.010) | (0.012) | (0.012) | (0.008) |
| L1 variation | 0.511^***^ | 0.626^***^ | 0.430^***^ | 0.701^***^ | 0.393^***^ |
|  | (0.007) | (0.009) | (0.006) | (0.010) | (0.005) |
| Observations | 16394 | 16685 | 16852 | 16520 | 16922 |

Unstandardized b-values; Standard errors in parentheses

^+^ *p* < 0.10, ^*^ *p* < 0.05, ^**^ *p* < 0.01, ^***^ *p* < 0.001

Controls for gender, age, and level of education

**Table E3.** The conditional effect of political distrust on support for decision-making processes, by internal efficacy, controlling for supporting government parties

|  | Delegate by electing parliament | Elected professional politicians | Direct democracy | Non-elected expert rule | Authoritarian |
| --- | --- | --- | --- | --- | --- |
| *Within* |  |  |  |  |  |
| Political distrust | -0.138^***^ | -0.282^***^ | 0.099^***^ | -0.075^***^ | -0.116^***^ |
|  | (0.012) | (0.013) | (0.011) | (0.014) | (0.010) |
| Importance of democracy | 0.073^***^ | 0.024^*^ | -0.028^**^ | -0.098^***^ | -0.107^***^ |
|  | (0.010) | (0.011) | (0.009) | (0.011) | (0.008) |
| Internal efficacy | 0.084^***^ | -0.017 | 0.044^***^ | -0.003 | -0.019^+^ |
|  | (0.013) | (0.014) | (0.012) | (0.015) | (0.011) |
| *Between* |  |  |  |  |  |
| Political distrust | -0.114^***^ | -0.428^***^ | 0.390^***^ | 0.025^+^ | 0.002 |
|  | (0.012) | (0.013) | (0.015) | (0.015) | (0.012) |
| Importance of democracy | 0.109^***^ | 0.019 | -0.086^***^ | -0.195^***^ | -0.361^***^ |
|  | (0.011) | (0.012) | (0.014) | (0.013) | (0.011) |
| Internal efficacy | 0.421^***^ | -0.057 | -0.281^***^ | -0.159^**^ | -0.135^***^ |
|  | (0.039) | (0.044) | (0.051) | (0.049) | (0.041) |
| *Ref: Support government party* | |  |  |  |  |
| opposition | 0.046^*^ | -0.136^***^ | 0.176^***^ | 0.063^*^ | -0.098^***^ |
|  | (0.020) | (0.022) | (0.026) | (0.025) | (0.021) |
| don't know/no party | -0.064^**^ | -0.193^***^ | 0.073^*^ | -0.034 | -0.153^***^ |
|  | (0.025) | (0.027) | (0.032) | (0.031) | (0.026) |
| *Interaction* |  |  |  |  |  |
| Political distrust (within)  * Internal efficacy (within) | -0.026 | -0.077^**^ | -0.017 | -0.037 | -0.045^+^ |
|  | (0.026) | (0.029) | (0.027) | (0.031) | (0.024) |
| Political distrust (between)  * Internal efficacy (between) | -0.095^***^ | -0.022 | 0.087^***^ | 0.021 | 0.009 |
|  | (0.013) | (0.014) | (0.016) | (0.016) | (0.013) |
|  | (0.025) | (0.027) | (0.032) | (0.031) | (0.026) |
| *Constant* | *3.449^***^* | *4.707^***^* | *2.414^***^* | *3.933^***^* | *4.776^***^* |
|  | *(0.086)* | *(0.095)* | *(0.109)* | *(0.107)* | *(0.088)* |
| L2 variation | 0.204^***^ | 0.259^***^ | 0.506^***^ | 0.361^***^ | 0.288^***^ |
|  | (0.008) | (0.009) | (0.012) | (0.012) | (0.008) |
| L1 variation | 0.508^***^ | 0.626^***^ | 0.430^***^ | 0.701^***^ | 0.392^***^ |
|  | (0.007) | (0.009) | (0.006) | (0.010) | (0.005) |
| Observations | 16345 | 16633 | 16799 | 16476 | 16869 |

Unstandardized b-values; Standard errors in parentheses

^+^ *p* < 0.10, ^*^ *p* < 0.05, ^**^ *p* < 0.01, ^***^ *p* < 0.001

Controls for gender, age, level of education, and country

**Table E4.** The conditional effect of political distrust on support for decision-making processes, by populist party leaning, controlling for supporting government parties

|  | Delegate by electing parliament | Elected professional politicians | Direct democracy | Non-elected expert rule | Authoritarian |
| --- | --- | --- | --- | --- | --- |
| *Within* |  |  |  |  |  |
| Political distrust | -0.170^***^ | -0.225^***^ | 0.066 | 0.037 | -0.056 |
|  | (0.042) | (0.048) | (0.045) | (0.049) | (0.042) |
| Importance of democracy | 0.079^***^ | 0.015 | -0.036^***^ | -0.118^***^ | -0.109^***^ |
|  | (0.011) | (0.012) | (0.010) | (0.012) | (0.009) |
| *Between* |  |  |  |  |  |
| Political distrust | -0.307^***^ | -0.276^***^ | 0.252^***^ | 0.131^***^ | 0.067^*^ |
|  | (0.031) | (0.034) | (0.040) | (0.039) | (0.033) |
| Importance of democracy | 0.137^***^ | -0.013 | -0.107^***^ | -0.228^***^ | -0.402^***^ |
|  | (0.012) | (0.013) | (0.015) | (0.015) | (0.013) |
| Populist party leaning | -0.059^**^ | 0.050^*^ | 0.036 | 0.071^**^ | 0.051^*^ |
|  | (0.020) | (0.022) | (0.026) | (0.025) | (0.021) |
| *Ref: Support government party* | |  |  |  |  |
| opposition | 0.027 | -0.102^***^ | 0.046^+^ | 0.044 | -0.127^***^ |
|  | (0.022) | (0.024) | (0.028) | (0.027) | (0.023) |
| don't know/no party | -0.079^*^ | -0.121^***^ | 0.020 | -0.024 | -0.131^***^ |
|  | (0.031) | (0.034) | (0.040) | (0.039) | (0.033) |
| *Interaction* |  |  |  |  |  |
| Political distrust (within)  * Pop. Party leaning (between) | 0.004 | -0.013 | 0.010 | -0.026^*^ | -0.015^+^ |
|  | (0.009) | (0.010) | (0.010) | (0.011) | (0.009) |
| Political distrust (between)  * Pop. Party leaning (between) | 0.032^***^ | -0.027^***^ | 0.020^*^ | -0.023^**^ | -0.014^*^ |
|  | (0.006) | (0.007) | (0.008) | (0.008) | (0.007) |
| *Constant* | *3.750^***^* | *4.576^***^* | *2.540^***^* | *3.829^***^* | *4.751^***^* |
|  | *(0.129)* | *(0.142)* | *(0.164)* | *(0.162)* | *(0.136)* |
| L2 variation | 0.091^***^ | 0.158^***^ | 0.297^***^ | 0.146^***^ | 0.241^***^ |
|  | (0.014) | (0.020) | (0.021) | (0.021) | (0.019) |
| L1 variation | 0.216^***^ | 0.263^***^ | 0.517^***^ | 0.383^***^ | 0.322^***^ |
|  | (0.008) | (0.010) | (0.013) | (0.013) | (0.009) |
| Random slope | 0.473^***^ | 0.579^***^ | 0.348^***^ | 0.648^***^ | 0.332^***^ |
| Political distrust (within) | (0.008) | (0.010) | (0.006) | (0.011) | (0.006) |
| Observations | 13938 | 14139 | 14268 | 14036 | 14321 |

Unstandardized b-values; Standard errors in parentheses

^+^ *p* < 0.10, ^*^ *p* < 0.05, ^**^ *p* < 0.01, ^***^ *p* < 0.001

Controls for gender, age, level of education, and country

**F – Modeling the effects on support for political change as a dichotomy**

**Table F1.** Support for political change (dichotomized) by political distrust, REWB models

|  | Political change |
| --- | --- |
| *Within* |  |
| Political distrust | 0.644^***^ |
|  | (0.039) |
| Importance of democracy | -0.178^***^ |
|  | (0.036) |
| *Between* |  |
| Political distrust | 1.493^***^ |
|  | (0.031) |
| Importance of democracy | -0.364^***^ |
|  | (0.029) |
| *Country (ref: UK)* |  |
| NL | -1.094^***^ |
|  | (0.051) |
| SE | -0.044 |
|  | (0.053) |
| PT | 0.576^***^ |
|  | (0.064) |
| *Constant* | *-0.775^***^* |
|  | *(0.218)* |
| Observations | 17064 |

Standard errors in parentheses

^+^ *p* < 0.10, ^*^ *p* < 0.05, ^**^ *p* < 0.01, ^***^ *p* < 0.001

N=17,064

Controls for gender, age, and level of education

**Table F2.** Support for political change (dichotomized) by political distrust, REWB models per country

|  | Political change | | | |
| --- | --- | --- | --- | --- |
|  | UK | NL | SE | PT |
| *Within* |  |  |  |  |
| Political distrust | 1.107^***^ | 0.247^***^ | 0.440^**^ | 0.423^**^ |
|  | (0.063) | (0.059) | (0.143) | (0.143) |
| Importance of democracy | -0.296^***^ | -0.072 | 0.076 | -0.047 |
|  | (0.052) | (0.066) | (0.112) | (0.100) |
| *Between* |  |  |  |  |
| Political distrust | 1.167^***^ | 1.505^***^ | 2.023^***^ | 1.212^***^ |
|  | (0.060) | (0.053) | (0.071) | (0.075) |
| Importance of democracy | -0.226^***^ | -0.329^***^ | -0.406^***^ | -0.530^***^ |
|  | (0.054) | (0.048) | (0.069) | (0.079) |
| *Constant* | *0.276* | *-2.639^***^* | *-1.739^***^* | *1.500^**^* |
|  | *(0.405)* | *(0.355)* | *(0.526)* | *(0.573)* |
| Observations | 4851 | 4811 | 4350 | 3052 |

Standard errors in parentheses

^+^ *p* < 0.10, ^*^ *p* < 0.05, ^**^ *p* < 0.01, ^***^ *p* < 0.001

Controls for gender, age, and level of education

**G – COVID19**

Data collection for this project took place in the Fall and Winter of 2020-2021. In our article, we discuss how this timing – during the second wave of the COVID-19 pandemic, and at the early stages of the widespread roll-out of vaccines - might have affected some of our conclusions. In this appendix, we describe this context more extensively.

**Level of trust and the rally round the flag**

The level of trust in politics was particularly volatile during the first wave of the pandemic, as a consequence of the rally round the flag effect (cf. Jennings et al. 2020; Bol et al. 2020). This rally effect was at least in part driven by the fear of the external threat of the pandemic (Erhardt et al. 2021; Van der Meer et al. 2022). However, after the first wave of infections subsided in the late Spring of 2020, trust in government slowly began to decline. The dominant trend after March/April 2020 was downwards (cf Eurobarometer 92-95).


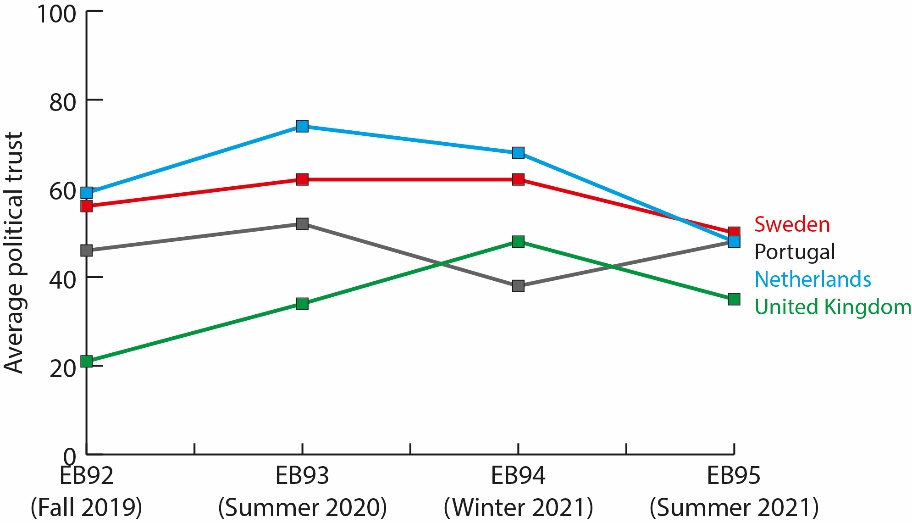


Figure G1. Average level of trust in government (source: Eurobarometer)

Our data basically focus on the time span between Summer 2020 and Winter 2021 (September-January/March), i.e., the second wave of infections. Just like the Eurobarometer data, we find different trends. In Portugal and the UK, we find a slight boost in trust/decrease in distrust (cf. Davies et al. 2021), whereas trends are less straightforward in Sweden and the Netherlands. There is thus no dominant trend. This is relevant to the external validity of our analyses.


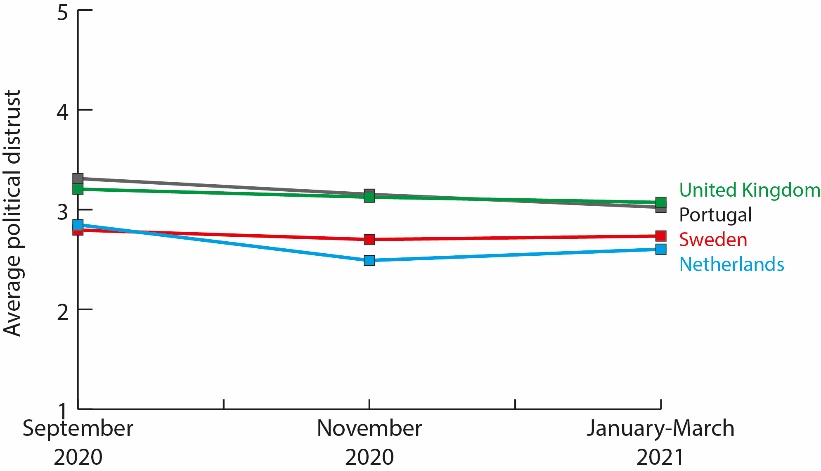


Figure G2. Average level of distrust in politics (source: own data)

**Excess mortality and anti-Covid measures**

The countries experienced similar but different trends in death tolls. Figure G3 visualizes the relative excess deaths (from all causes) per week by country from January 1^st^ 2020 until March 28^th^ 2021. It shows the rising death number of excess deaths in all countries between wave 1 (September) and wave 2 (November); whereas we find differential trends from circa January 2021 onwards.

Concurrently, COVID-measures showed relatively little variation between waves 1 and 2 of our data, as Figure G4 shows on the basis of the Oxford COVID-19 Government Response Tracker (Hale et al. 2021). However, after wave 2 (November) policies became stricter in all four countries, with some variation: relatively stable in Sweden, particularly strict in the United Kingdom, and more variable in Portugal. It would nevertheless be rather difficult to relate particular responses in the strictness of measures to the outcomes we find in our article. There is no evident pattern that might explain cross-national variation in support for (or rejection of) authoritarianism or elite rule.


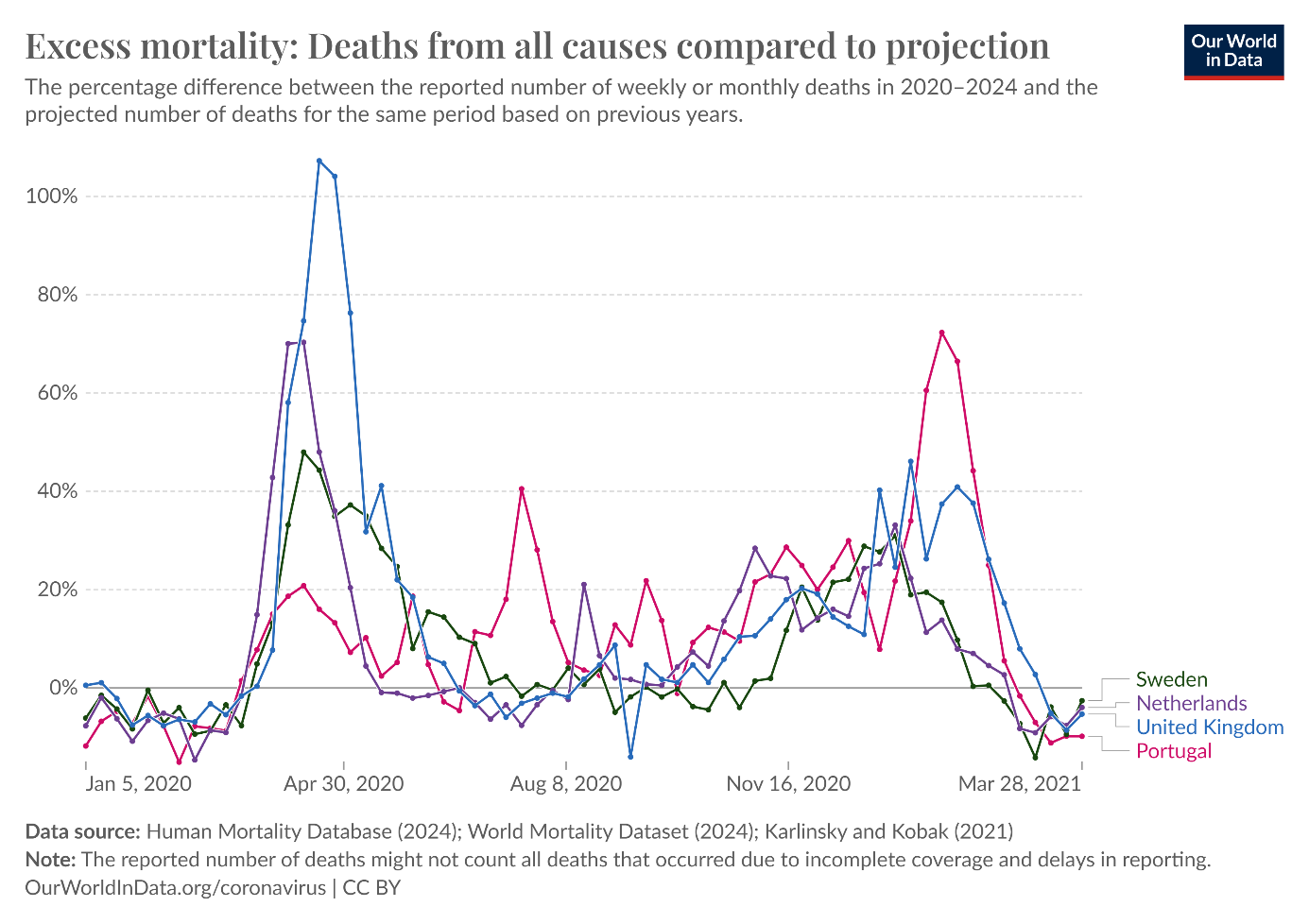


Figure G3. Excess mortality per week by country (source: OurWorldInData.org)


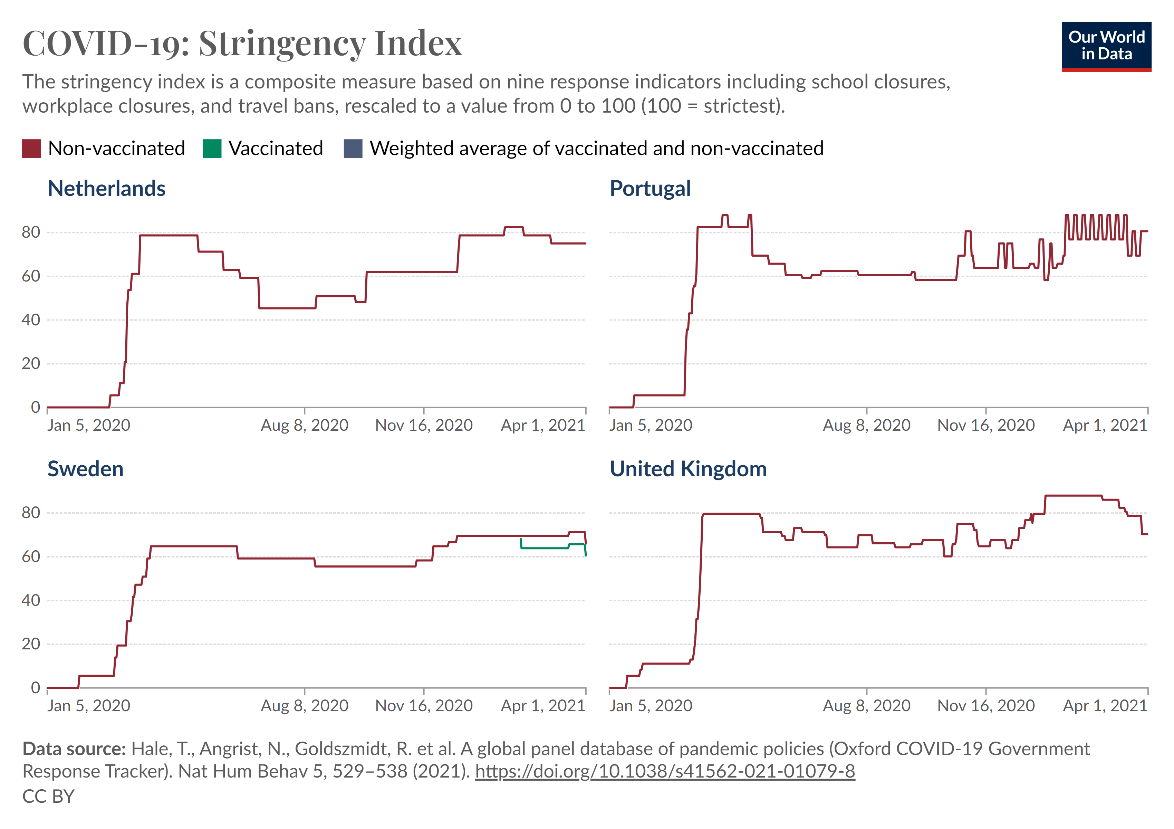


Figure G4. Stringency of COVID-19 government measures (source: Hale et al. 2021; OurWorldInData.org)

**COVID-19, technocracy, and authoritarianism**

Finally, it is unclear to what extent the pandemic affected support for alternative decision-making models, and particularly technocracy and authoritarianism. COVID-19 stimulated both populist and technocratic responses in politics (Esmark 2021). Yet, public opinion is less straightforward.

If anything, support for expert rule is most likely to be affected by the pandemic. Cena and Roccato (2023) found that both the contextual prevalence of COVID-19 and subjective vulnerability to COVID-19 stimulated support for technocracy; the latter particularly among those who distrust parliament. Lavezzolo et al. (2023), however, conclude that “the increase in technocratic attitudes [during the pandemic] is driven by a growth of pro-expertise preferences, while the elitist and anti-politics components do not change”, but also that technocratic attitudes are related to distrust towards government. Yet, the within-person effects in our study (see Table 3 in the article) go in the opposite direction: rising political distrust tends to lower support for expert rule. To the extent that COVID had an effect, the between-person studies discussed above suggest that the effect we find might be a conservative estimate. Alternatively, during the pandemic the trusters rather than the distrusters may have been more likely to support expert rule, in line with the technocratic response inherent in policy responses.

Concurrently, recent studies suggest that authoritarian attitudes were stimulated by COVID-19 (Hirsch 2022) and the COVID-19 lockdown measures (Marbach et al. 2021). These studies do not necessarily indicate rising support for authoritarianism as a rivaling decision-making model, but do indicate support for the underlying attitudes. Moreover, it is not clear in the literature to what extent distrust affects this relationship.

In the end, it is not clear to what extent and even in which direction the findings in our study might be affected by its timing during the second wave of the COVID19-pandemic. Evidence suggests that effects may be relatively moderate. Yet, any inference is complicated, as earlier studies tended to conflate the between- and within-person effects of political trust.

**References**

Bol, D., Giani, M., Blais, A., and Loewen, P.J. (2021). The Effect of COVID-19 Lockdowns on Political Support: Some Good News for Democracy?. *European Journal of Political Research*, *60*(2), 497–505.

Cena, L., and Roccato, M. (2023). Desires of technocracy in pandemic times: A multilevel study. *Social Science Quarterly, 104*(3), 195-201.

Davies, B., Lalot, F., Peitz, L., Heering, M.S., Ozkececi, H., Babaian, J., Davies Hayon, K., Broadwood, J. & Abrams, D. (2021). Changes in political trust in Britain during the COVID-19 pandemic in 2020: integrated public opinion evidence and implications. *Humanities and Social Sciences Communications*, 8: 166.

Erhardt, J., Freitag, M., Filsinger, M., and Wamsler, S. (2021). The Emotional Foundations of Political Support: How Fear and Anger Affect Trust in the Government in Times of the Covid-19 Pandemic. *Swiss Political Science Review*, *27*(2), 339–352.

Esmark, A. (2023). How does crisis affect the conflict between technocracy and populism? Lessons from the COVID-19 pandemic. *Politics*, *43*(4), 520-535.

Hirsch, M. (2022). Becoming authoritarian for the greater good? Authoritarian attitudes in context of the societal crises of COVID-19 and climate change. *Frontiers in Political Science*, 4. DOI: 10.3389/fpos.2022.929991.

Jennings, W., Valgardsson, V., Stoker, G., Devine, D., Gaskell, J., and Evans, M. (2020). Political Trust and the COVID-19 Crisis: Pushing Populism to the Backburner? A Study of Public Opinion in Australia, Italy, the UK and the USA. *TrustGov Project, Democracy 2025*, available at <https://www.ipsos.com/sites/default/files/ct/news/documents/2020-08/covid_and_trust.pdf>

Lavezzolo, S., Ramiro, L., and Fernández-Vázquez, P. (2022). Technocratic attitudes in COVID-19 times: Change and preference over types of experts. *European Journal of Political Research*, *61*(4), 1123-1142.

Marbach, M., Ward, D., and Hangartner, D. (2021) Do COVID-19 Lockdown Policies Weaken CivicAttitudes? Survey Evidence from the United States and Europe. *SocArXiv*, DOI: 10.31235/osf.io/5nsgc

Van der Meer, T.W.G., Steenvoorden, E.J. & Ouattara, E.M. (2023). Fear and the COVID-19 rally round the flag: a panel study on political trust. *West European Politics*, DOI:

10.1080/01402382.2023.2171220.
